# Iodine and Nickel Ions Adsorption by Conjugated Copolymers Bearing Repeating Units of Dicyclopentapyrenyl and Various Thiophene Derivatives

**DOI:** 10.3390/polym15204153

**Published:** 2023-10-19

**Authors:** Suchetha Shetty, Noorullah Baig, Sk Abdul Wahed, Atikur Hassan, Neeladri Das, Bassam Alameddine

**Affiliations:** 1 Department of Mathematics and Natural Sciences, Gulf University for Science and Technology, Mubarak Al-Abdullah 32093, Kuwait; 2 Functional Materials Group, Gulf University for Science and Technology, Mubarak Al-Abdullah 32093, Kuwait; 3 Department of Chemistry, Indian Institute of Technology Patna, Patna 801106, Bihar, Indiaatikurhassan@gmail.com (A.H.); neeladri@iitp.ac.in (N.D.)

**Keywords:** cyclopolymerization, conjugated copolymers, iodine adsorption, heavy metal ion adsorption, environmental remediation

## Abstract

The synthesis of three conjugated copolymers TPP1–3 was carried out using a palladium-catalyzed [3+2] cycloaddition polymerization of 1,6-dibromopyrene with various dialkynyl thiophene derivatives 3a–c. The target copolymers were obtained in excellent yields and high purity, as confirmed by instrumental analyses. TPP1–3 were found to divulge a conspicuous iodine adsorption capacity up to 3900 mg g^−1^, whereas the adsorption mechanism studies revealed a pseudo-second-order kinetic model. Furthermore, recyclability tests of TPP3, the copolymer which revealed the maximum iodine uptake, disclosed its efficient regeneration even after numerous adsorption-desorption cycles. Interestingly, the target copolymers proved promising nickel ions capture efficiencies from water with a maximum equilibrium adsorption capacity (q_e_) of 48.5 mg g^−1^.

## 1. Introduction

The growing interest in conjugated polymers as attractive materials for various applications, notably organic electronic devices, is due to their broad absorption of light, modular emission, and charge transport capacity [1,2]. Synthesis and applications of various polycondensed aromatic hydrocarbons (PAHs) bearing heteroatoms, especially sulfur, have received remarkable attention due to their unique photophysical characteristics, low bandgaps, and superior chemical stability against photo-degradation and oxidation [3,4,5]. Cyclopentannulated polycyclic aromatic hydrocarbons (CP-PAHs) are considered a prominent class of conjugated polymers due to their extensive conjugation network, hence allowing for their use in optoelectronic applications, namely, organic photovoltaic devices (OPVs) and organic field-effect transistors (OFETs) [6,7,8], besides their possible use in chemotherapy as DNA intercalators thanks to their mutagenic and carcinogenic activities [9]. Recently, our group has reported the efficient synthesis of various cyclopentannulated conjugated copolymers, which were employed for the first time as adsorbents of iodine and the carcinogenic Basic Red 9 dye and iodine from the aqueous solution [10,11].

Nuclear power is considered an eminent energy source to produce electricity at a large scale in order to meet the great demand of an increasing global population and growing economy [12]. Nevertheless, the release of radioactive nuclear waste, like ^90^Sr, ^99^Tc, ^137^Cs, ^129^I, ^127^Xe, ^85^Kr, etc., is considered to be among the chief drawbacks of producing electricity from nuclear power [13,14,15]. Radioiodine, which occurs in the form of ^131^I (t_1/2_ = 8 days) and ^129^I (t_1/2_ = 15.7 million years), is the main gaseous pollutants causing severe health effects on humans and damaging the environment [16,17,18], where both wet scrubbing and physical adsorption are the most commonly employed techniques to filter off radioiodine vapors [19,20], with adsorption by porous materials being considered the most advantageous due to several factors, namely, their structural variety, cost-effectiveness, superior stability, and the possibility to engineer their porosity [21,22,23]. Therefore, various iodine adsorbents have been developed, among others, organic [24,25], inorganic [26], and hybrid [27] polymer networks, with the former (i.e., organic-based materials) as the ideal candidates for iodine capture, given their ease of synthesis, structural diversity, porosity, and stability [28].

Heavy metal ions, such as Mn(II), Co(II), Ni(II), and Cu(II), which are discharged into the aquatic system from various industrial activities like mining, metal plating, battery manufacturing, welding, and alloy manufacturing [29,30,31], have been considered a major environmental challenge over the past few years because they are non-biodegradable, leading to their accumulation in the atmosphere and living organisms on the one hand, in addition to their high toxicity, which causes acute human health hazards on the other hand [32]. Nickel, in particular, can cause adverse health complications to the lungs, kidneys, mucous membranes, as well as the reproductive and nervous systems [33]. Several separation techniques have been developed to remove heavy metal ions from aqueous media, such as precipitation [34,35], ion exchange [36], membranes [37], and adsorption [38], with the latter being widely employed because of its simplicity and cost-effectiveness [39]. In this work, we report the synthesis of new conjugated copolymers bearing repeating units of dicyclopenta[cd,jk]pyrenyl units and thiophene derivatives using a versatile palladium-catalyzed cyclopentannulation reaction for the efficient removal of iodine from the air and nickel ions from water.

## 2. Materials and Methods

All the reactions were carried out under an inert atmosphere of dry argon, and the chemical reagents were utilized as acquired from Merck (Darmstadt, Germany), and HiMedia (Mumbai, India) unless otherwise specified. The solvents were deoxygenated by bubbling with a positive flux of dry argon for 30 min. Thin-layer chromatography (TLC) was carried out on aluminum sheets coated with silica gel 60 F254 and revealed using a UV lamp. ^1^H- and ^13^C- nuclear magnetic resonance (NMR) spectra were recorded utilizing a Bruker 400 MHz (Mannheim, Germany) NMR instrument by dissolving the respective compounds in CDCl_3_ at 25 °C. Chemical shifts (in ppm) are reported using tetramethylsilane (δ = 0) as an internal standard in either CDCl_3_ or CD_2_Cl_2_ solvent. Thermogravimetric analysis (TGA) was recorded on a Shimadzu TGA-60H (Kyoto, Japan) analyzer and was employed to measure the thermal stability of the copolymers from room temperature up to 800 °C with a 10 °C/min heating rate under an inert atmosphere. UV–Vis spectra were recorded using a Shimadzu UV1800 spectrophotometer, whereas fluorescence spectra were recorded on an Agilent G9800 Cary Eclipse Fluorescence spectrophotometer (Santa Clara, CA, USA). Brunauer–Emmett–Teller (BET) surface area and porosity measurements were measured using a surface area and pore size analyzer (Gemini-V, Micromeritics, Norcross, GA, USA) at the boiling point of liquid nitrogen (−196 °C). Samples were degassed overnight before the experiments in the VacuPrep 061 sample degassing system at a temperature of 105 °C. Surface areas (SBET) were calculated using the Brunauer–Emmet–Teller (BET) model of isotherms and the adsorption of N_2_ at small relative pressures. Total pore volume (Vt) was determined from the specific adsorption of N_2_ at a p/p^0^ = 0.99. The t-plot method was used to estimate micropore volume (V_mic_) and external surface area (S_ext_). A Quantachrome autosorb iQ2 analyzer was used to collect gas adsorption data. In a typical gas uptake setup, 120–150 mg of a given sample were loaded in a 9 mm cell and were degassed at 120 °C for 5–8 h. The cells with the degassed materials were refilled with Helium gas and accurately weighed before they were reattached to the analysis unit of the instrument for measurements. Various temperatures of the analysis unit sample cell were maintained using a KGW isotherm bath that was filled with liquid N_2_ (77 K) or using a temperature-controlled bath (298 K and 273 K). The molar masses of the polymers were recorded on an Agilent 1260 infinity II gel permeation chromatograph (GPC) against a refractive index (RI) detector at room temperature and two columns (PL mixed-C), which are calibrated against twelve monodisperse polystyrene (PS) standards with THF employed as the eluent at a flow rate of 1.0 mL min^−1^.

### 2.1. Synthesis

#### 2.1.1. Synthesis of 3a (Procedure A)

2,5-dibromo-3-hexylthiophene 2a (0.7 g, 2.14 mmol, 1 eq.), 1-(tert-butyl)-4-ethynylbenzene 1a (1.59 g, 10.1 mmol, 4.7 eq.), Pd_2_(dba)_3_ (12 mg, 0.013 mmol, 0.6 mol%), PPh_3_ (50 mg, 0.19 mmol), and CuI (8.2 mg, 0.043 mmol) were added to a Schlenk tube containing 18 mL of degassed diisopropylamine (iPr_2_NH) and the mixture was heated at 90 °C for 2 days under argon. The solvent was evaporated under reduced pressure, and the resulting residue was extracted with DCM from a saturated solution of NaHCO_3_ (100 mL). The organic layer was then washed with deionized water (75 mL × 2), followed by evaporation of the solvent. The desired product was purified by silica gel column chromatography using petroleum ether/DCM (90:10 *v*/*v*) as the eluent, yielding a pale yellow solid (950 mg, 95%). ^1^H-NMR (400 MHz, CD_2_Cl_2_, ppm): δ 7.41–7.39 (m, 4H, ArH), 7.38–7.36 (m, 4H, ArH), 7.06 (s, 1H, ArH), 2.73 (t, 2H, -CH_2_), 1.67 (t, 2H, -CH_2_), 1.38 (s, 2H, -CH_2_), 1.34 (s, 18H, -CH_3_), 1.29 (s, 4H, -CH_2_), and 0.91 (t, 3H, -CH_3_). ^13^C-NMR (400 MHz, CD_2_Cl_2_, ppm): δ 152.10, 132.74, 131.88, 131.21, 125.50, 125.34, 123.04, 119.06, 83.82, 34.87, 31.66, 31.16, 30.11, 29.52, 28.91, 22.63 and 14.14. EI-HRMS: *m*/*z* calculated for M^•+^ C_34_H_40_S 480.2851 found 480.1027. FTIR (KBr, cm^−1^): 2959 (Aliphatic C-H str), 2197 (CΞC Str), 1509 (Aromatic C=C str), 1460 (Aliphatic C-H ben), 831 (Aromatic C-H ben), and 730 (C-S str).

#### 2.1.2. Synthesis of 3b

3b was prepared following Procedure A with 2,6-dibromodithieno [3,2-b:2′,3′-d]thiophene 2b (0.5 g, 1.4 mmol, 1 eq.), 1-(tert-butyl)-4-ethynylbenzene 1a (1 g, 6.6 mmol, 4.7 eq.) Pd_2_(dba)_3_ (7.8 mg, 0.009 mmol, 0.6 mol%), PPh_3_ (33 mg, 0.12 mmol), and CuI (5.4 mg, 0.03 mmol) in 20 mL of degassed diisopropylamine (iPr_2_NH) at 90 °C for 2 days. It was isolated by column chromatography using petroleum ether/DCM (90:20 *v*/*v*) as the eluent. Pale pink solid (702 mg, 97%). ^1^H-NMR (400 MHz, CD_2_Cl_2_, ppm): δ 7.44–7.43 (m, 4H, ArH), 7.40–7.38 (m, 4H, ArH), 7.20 (m, 1H, ArH), 7.17 (m, 1H, ArH), and 1.33 (s, 18H, -CH_3_). ^13^C-NMR (400 MHz, CD_2_Cl_2_, ppm): δ 152.21, 141.69, 131.29, 128.57, 125.58, 125.03, 124.48, 119.51, 95.27, 82.23, 34.97, and 31.24. EI-HRMS: *m*/*z* calculated for M^•+^ C_32_H_28_S_3_ 508.1353 found 508.8241. FTIR (KBr, cm^−1^): 2964 (Aliphatic C-H str), 2202 (CΞC Str), 1524 (Aromatic C=C str), 1460 (Aliphatic C-H ben), 831 (Aromatic C-H ben), and 693 (C-S str).

#### 2.1.3. Synthesis of 3c

3c was prepared following Procedure A with 5,5″-dibromo-2,2′:5′,2″-terthiophene 2c (0.5 g, 1.2 mmol, 1 eq.), 1-(tert-butyl)-4-ethynylbenzene 1a (0.8 g, 5.2 mmol, 4.7 eq.) Pd_2_(dba)_3_ (6 mg, 0.0067 mmol, 0.6 mol%), PPh_3_ (26 mg, 0.1 mmol), and CuI (4.2 mg, 0.02 mmol) in 18 mL of degassed diisopropylamine (iPr_2_NH) at 90 °C for 2 days. Purified by column chromatography using petroleum ether/DCM (90:20 *v/v*) as the eluent. Pale pink solid (657 mg, 95%). ^1^H-NMR (400 MHz, CD_2_Cl_2_, ppm): δ 7.68 (m, 2H, ArH), 7.41 (m, 8H, ArH), 7.33–7.29 (m, 4H, ArH), 1.33 (s, 18H, -CH_3_). ^13^C-NMR (400 MHz, CD_2_Cl_2_, ppm): δ 152.20, 138.45, 132.70, 130.83, 131.21, 125.53, 124.74, 124.00, 123.73, 119.73, 94.79, 82.01, 34.94, and 31.24. EI-HRMS: *m*/*z* calculated for M^•+^ C_36_H_32_S_3_ 560.1666 found 560.3068. FTIR (KBr, cm^−1^): 2964 (Aliphatic C-H str), 2197 (CΞC Str), 1509 (Aromatic C=C str), 1468 (Aliphatic C-H ben), 794 (Aromatic C-H ben), and 574 (C-S str).

#### 2.1.4. Synthesis of 3d

3d was prepared following Procedure A with 2-bromo-5-hexylthiophene 2d (0.3 g, 1.2 mmol, 1 eq.), 1-(tert-butyl)-4-ethynylbenzene 1a (0.46 g, 2.9 mmol, 2.4 eq.), Pd_2_(dba)_3_ (6.7 mg, 0.0073 mmol, 0.6 mol%), PPh_3_ (28 mg, 0.1 mmol), and CuI (4.6 mg, 0.02 mmol) in 10 mL of degassed diisopropylamine (iPr_2_NH) at 90 °C for 2 days. It was isolated by column chromatography using petroleum ether/DCM (90:10 *v*/*v*) as the eluent. Pale pink solid (386 mg, 98%). ^1^H-NMR (400 MHz, CD_2_Cl_2_, ppm): δ 7.42 (d, 2H, ArH), 7.37 (d, 2H, ArH), 7.08 (d, 1H, ArH), 6.67 (d, 1H, ArH), 2.79 (t, 2H, -CH_2_), 1.67 (t, 2H, -CH_2_), 1.32 (s, 9H, -CH_3_), 1.31 (s, 4H, -CH_2_), 1.30 (s, 2H, -CH_2_), and 0.89 (t, 3H, -CH_3_). ^13^C-NMR (400 MHz, CD_2_Cl_2_, ppm): δ 151.47, 148.12, 131.88, 131.08, 125.36, 124.17, 120.76, 120.14, 119.06, 92.43, 83.83, 34.81, 31.57, 31.19, 31.16, 30.25, 28.73, 22.60, and 14.11. EI-HRMS: *m*/*z* calculated for M^•+^ C_22_H_28_S 324.1912 found 324.1021. FTIR (KBr, cm^−1^): 2964 (Aliphatic C-H str), 2207 (CΞC Str), 1509 (Aromatic C=C str), 1464 (Aliphatic C-H ben), 836 (Aromatic C-H ben), and 570 (C-S str).

#### 2.1.5. Synthesis of TPM

A Schlenk tube was charged with 1,6-dibromopyrene DBP (0.1 g, 0.28 mmol,1 eq.), 2-((4-(tert-butyl)phenyl)ethynyl)-5-hexylthiophene 3d (0.2 g, 0.61 mmol, 2.2 eq.), Pd_2_(dba)_3_ (25 mg, 0.028 mmol, 10 mol%), tris(o-tolyl)phosphine P(o-tol)_3_, (8.5 mg, 0.028 mmol), KOAc (0.13 g, 1.38 mmol), and LiCl (24 mg, 0.56 mmol) in 8 mL 1:1 *v*/*v* mixture of a degassed DMF/toluene under argon. The reaction mixture was heated at 130 °C for 24 h and the solvent was removed under reduced pressure. The resulting residue was dissolved in DCM and extracted with a saturated NaHCO_3_ solution (50 mL × 2). The combined organic layer was washed with deionized water (75 mL × 2), then the solvent was removed, and the desired product was isolated by silica gel column chromatography using petroleum ether/DCM (90:10 *v*/*v*) as eluent. Brick red solid (219 mg, 93%).^1^H-NMR (400 MHz, CD_2_Cl_2_, ppm): δ 8.08 (m, 2H, ArH), 7.84–7.82 (d, 2H, ArH), 7.76–7.63 (m, 4H, ArH), 7.52–7.47 (m, 6H, ArH), 7.41 (d, 2H, ArH), 6.97 (s,2H, ArH) 2.82 (t, 4H, -CH_2_), 1.75 (s, 4H, -CH_2_), 1.34–1.30 (m, 20H, -CH_2_,CH_3_), 1.39 (s, 6H, -CH_2_), 1.26 (s, 4H, -CH_2_), and 0.88 (t, 6H, -CH_3_). ^13^C-NMR (400 MHz, CD_2_Cl_2_, ppm): δ 152.95, 145.87, 141.05, 140.89, 138.31, 136.94, 134.64, 133.69, 129.85, 129.19, 128.44, 127.65, 126.41, 126.27, 125.87, 125.40, 125.27, 122.29, 34.75, 31.58, 31.41, 30.33, 28.86, 22.63, and 14.16. EI-HRMS: *m*/*z* calculated for M^•+^ C_60_H_62_S_2_ 846.4293 found 846.4260. FTIR (KBr, cm^−1^): 2959 (Aliphatic C-H str), 1509 (Aromatic C=C str), 1460 (Aliphatic C-H ben), 836 (Aromatic C-H ben), and 684 (C-S str).

#### 2.1.6. Synthesis of TPP1 (Procedure B)

2,5-bis((4-(tert-butyl)phenyl)ethynyl)-3-hexylthiophene 3a (200 mg, 0.42 mmol, 1 eq.), 1,6-dibromopyrene DBP (150 mg, 0.42 mmol, 1 eq.), Pd_2_(dba)_3_ (38 mg, 0.042 mmol, 10 mol%.), P(o-tol)_3_ (19 mg, 0.063 mmol), KOAc (204 mg, 2.08 mmol), and LiCl (35 mg, 0.83 mmol) were charged in a Schlenk tube and heated at 130 °C in 8.2 mL of a 1:1 DMF/toluene mixture under argon for 5 days. The solvent was completely removed under reduced pressure, and the residue was dissolved in DCM and extracted with a saturated solution of NaHCO_3_ (50 mL × 2). The organic layer was washed with deionized water (100 mL × 3), concentrated, and precipitated from petroleum ether. The green solid was filtered and washed excessively with petroleum ether, acetone, and methanol and then dried under vacuum. Brick red solid (267 mg, 94%).^1^H-NMR (400 MHz, CD_2_Cl_2_, ppm): δ 7.99–7.42 (m, 15H, ArH), 2.07 (br, 2H, -CH_2_), and 1.31 (br, 29H, -CH_2_, -CH_3_). ^13^C-NMR (400 MHz, CD_2_Cl_2_, ppm): δ 146.00, 141.96, 141.37, 138.09, 137.82, 134.77, 132.38, 130.81, 130.05, 129.47, 126.94, 125.46, 125.30, 125.16, 124.94, 123.62, 120.41, 31.49, 29.79, 22.41, and 16.12. FTIR (KBr, cm^−1^): 2959 (Aliphatic C-H str), 1650 (Aromatic C=C str), 1465 (Aliphatic C-H ben), 836 (Aromatic C-H ben), and 689 (C-S str). GPC (THF): Mw (KDa): 12.28, Mn (KDa): 6.18, Ð: 1.98.

#### 2.1.7. Synthesis of TPP2

TPP2 was prepared following Procedure B with 2,6-bis((4-(tert-butyl)phenyl)ethynyl)dithieno[3,2-b:2′,3′-d]thiophene 3b (200 mg, 0.39 mmol, 1 eq.), 1,6-dibromopyrene DBP (142 mg, 0.39 mmol, 1 eq.), Pd_2_(dba)_3_ (36 mg, 0.039 mmol, 10 mol%), P(o-tol)_3_ (18 mg, 0.059 mmol), KOAc (193 mg, 1.97 mmol), and LiCl (33 mg, 0.79 mmol) in 7.8 mL of a 1:1 DMF/toluene mixture under argon at 130 °C for 5 days. Brick red solid (262 mg, 95%). ^1^H-NMR (400 MHz, CD_2_Cl_2_, ppm): δ 7.95 (m, 8H, ArH), 7.66 (m, 2H, ArH), 7.47 (m, 2H, ArH), 7.36 (m, 2H, ArH), 7.13 (m, 2H, ArH), and 1.39 (br, 18H, -CH3). FTIR (KBr, cm^−1^): 2954 (Aliphatic C-H str), 1647 (Aromatic C=C str), 1450 (Aliphatic C-H ben), 840 (Aromatic C-H ben), and 693 (C-S str).

#### 2.1.8. Synthesis of TPP3

TPP3 was prepared following Procedure B with 5,5″-bis((4-(tert-butyl)phenyl)ethynyl)-2,2′:5′,2″-terthiophene 3c (200 mg, 0.36 mmol, 1 eq.), 1,6-dibromopyrene DBP (129 mg, 0.36 mmol, 1 eq.), Pd_2_(dba)_3_ (33 mg, 0.036 mmol, 10 mol%), P(o-tol)_3_ (16 mg, 0.053 mmol), KOAc (175 mg, 1.78 mmol), and LiCl (30 mg, 0.71 mmol) in 7 mL of a 1:1 DMF/toluene mixture under argon at 130 °C for 5 days. Brick red solid (263 mg, 96%). ^1^H-NMR (400 MHz, CD_2_Cl_2_, ppm): δ 7.95 (m, 10H, ArH), 7.40 (br, 2H, ArH), 7.28 (m, 4H, ArH), 7.06 (br, 4H, ArH), and 1.36 (br, 18H, -CH_3_). FTIR (KBr, cm^−1^): 2927 (Aliphatic C-H str), 1601 (Aromatic C=C str), 1450 (Aliphatic C-H ben), 843 (Aromatic C-H ben), and 702 (C-S str).

### 2.2. Iodine Uptake Studies of TPP1–3

A 10 mg sample of a given target copolymer TPP1–3 and iodine flakes were kept in two connected and closed pre-weighed bottles. The vessel was degassed and maintained at 80 °C, and the iodine uptake was measured at different time intervals. The wt.% of iodine adsorbed by the conjugated polymer was calculated using the following equation:

Iodine uptake = (M_2_ − M_1_)/M_1_ × 100 wt.% (100 wt.% = 1000 mg g^−1^) (1)

where M_1_ and M_2_ are the respective masses of the copolymer before and after iodine adsorption [40].

#### Adsorption Equilibrium and Kinetics

The adsorption kinetics were analyzed using the pseudo-first-order and pseudo-second-order models, as expressed in Equations (2) and (3) below:

ln (q_e_ − q_t_) = ln q_e_ − k_1_t (2)

t/q_t_ = t/q_e_ + 1/k_2_q_e_^2^ (3)

where q_t_ (mg g^−1^) and q_e_ (mg g^−1^) denote the amounts of iodine absorbed per gram adsorbent at time t and at equilibrium, respectively. k_1_ and k_2_ represent the rate constants of the pseudo-first-order model and pseudo-second-order model, respectively [41,42].

### 2.3. Nickel Ion Adsorption Studies of TPP1–3

TPP1–3 were tested as nickel ion adsorbents by stirring 5 mg of a given copolymer sample into a 5 mL aqueous solution of Ni^2+^ with an initial concentration of 10–200 mg L^−1^ overnight at room temperature. The Ni^2+^ concentration in the solution before and after the adsorption by TPP1–3 was measured using a UV–Vis absorption spectrophotometer, and the equilibrium adsorption capacity, q_e_ (mg g^−1^), of TPP1–3 for nickel was determined using the following equation:

q_e_(mg g^−1^) = (C_o_ − C_e_) V/m (4)

where C_o_ and C_e_ are the initial and equilibrium concentrations of the Ni^2+^ aqueous solutions (mg L^−1^), respectively. m (g) is the quantity of the adsorbent TPP1–3 that was used, and V (L) is the volume of the Ni^2+^ solution [43].

## 3. Results and Discussion

**Scheme 1:**
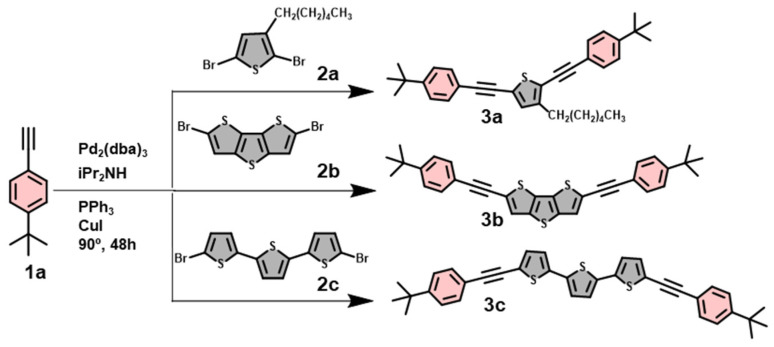
Synthesis of comonomers 3a–c.

Scheme 1 depicts the synthesis of the diethynyl thiophene surrogates 3a–c, which employs a conventional palladium-catalyzed Sonogashira cross-coupling reaction by reacting 1-(tert-butyl)-4-ethynylbenzene 1a with various dibrominated thiophene-based derivates 2a–c. The desired comonomers 3a–c were isolated in high yields (95–97%) from the reaction medium by silica gel column chromatography using petroleum ether/DCM (90:10 *v*/*v*) as the eluent. The structure and purity of 3a–c were confirmed by ^1^H- and ^13^C- NMR, EI-MS, and FTIR spectroscopy (Figures S1–S3, S9–S11, S15–S17 and S23–S25 in the Supplementary Materials).

**Scheme 2:**
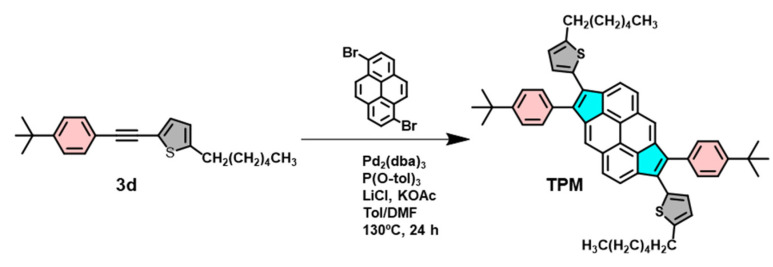
Synthesis of monomer TPM.

**Figure 1:**
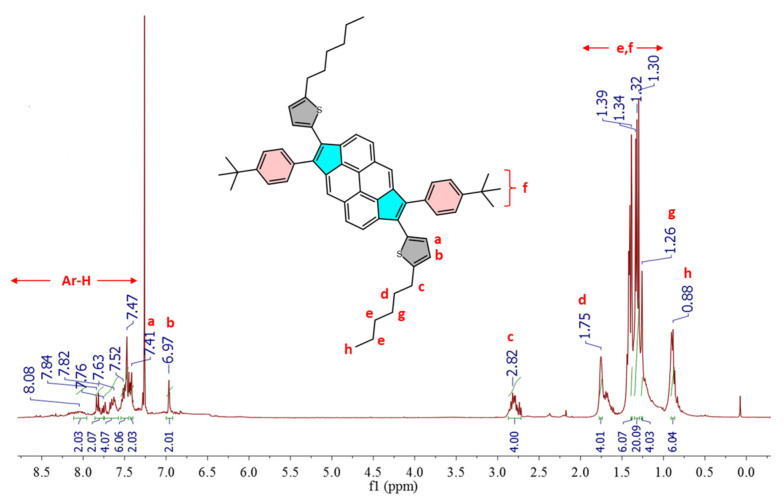
^1^H-NMR spectrum of TPM recorded in CDCl_3_.

The prototypical monomer TPM was prepared following reaction conditions to test the viability of the reaction, which is similar to those we have previously reported in the literature [10]. Thus, 1,6-dibromopyrene DBP was reacted with two equivalents of 2-((4-(tert-butyl)phenyl)ethynyl)-5-hexylthiophene 3d in the presence of pd_2_(dba)_3_ catalyst, P(o-tol)_3_ ligand, KOAc, and LiCl under dry argon in a degassed 1:1 mixture of DMF/toluene at 130 °C for 24 h, which afforded the desired product in 93% yield (Scheme 2) and high purity, as corroborated by ^1^H- and ^13^C- NMR, ESI-MS, and FTIR spectroscopy (Figure 1 and Figures S5, S13, S19, and S27 in the Supplementary Materials).

**Scheme 3:**
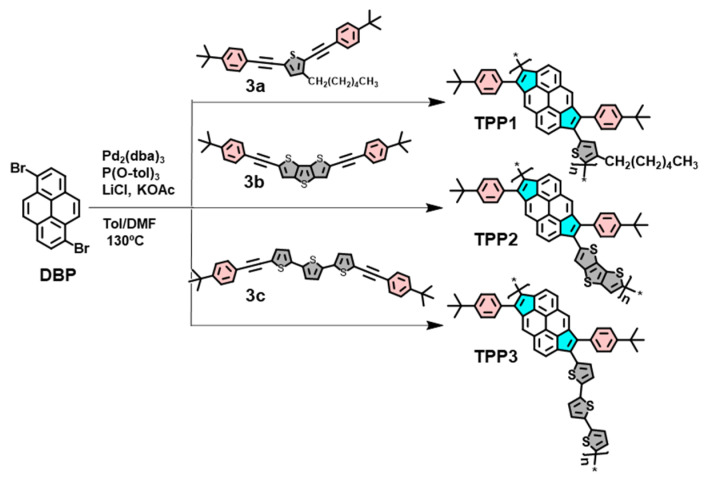
Synthesis of conjugated polymers TPP1–3.

The conjugated copolymers TPP1–3 were synthesized employing similar reaction conditions to those depicted in Scheme 2 to prepare the prototypical monomer TPM. Therefore, Scheme 3 reveals the synthesis of TPP1–3 which were made from the [3+2] cyclocondensation polymerization reaction of synthon DBP with three different dialkynyl thiophene derivatives, namely, 2,5-bis((4-(tert-butyl)phenyl)ethynyl)-3-hexylthiophene 3a, 2,6-bis((4-(tert-butyl)phenyl)ethynyl)-dithieno[3,2-b:2′,3′-d]thiophene 3b, and 5,5″-bis((4-(tert-butyl)phenyl)ethynyl)-2,2′:5′,2″-terthiophene 3c. The hitherto mentioned reaction afforded TPP1–3 in excellent yields (>93%) after their isolation from the reaction mixture by extraction using DCM, followed by their precipitation from a DCM–petroleum ether mixture, and before the filtered precipitates underwent exhaustive washings with petroleum ether, acetone, and methanol. The resulting conjugated copolymer TPP1, i.e., the one bearing n-hexyl chains, was found to be highly soluble in common organic solvents such as DCM, CHCl_3_, and THF. On the other hand, copolymers TPP2,3 were found to be partially soluble in common organic solvents like DCM, CHCl_3_ and THF. The structures of copolymers TPP1–3 were analyzed by ^1^H- and ^13^C- NMR, FTIR, and UV-emission spectroscopies.

Figure 1 below portrays the ^1^H-NMR spectrum of prototypical monomer TPM in CDCl_3_, where the chemical shifts in the range of 8.08–7.47 ppm are assigned to the 14 aromatic protons of the target monomer, whereas the two protons at 7.41 ppm and two protons at 6.97 ppm are attributed to the protons of the thiophene rings. Fourty-four protons observed in the aliphatic region extending from 2.82 to 0.88 ppm are correlated to the hexyl and t-butyl units. Furthermore, the ^13^C-NMR spectrum of TPM illustrates all the characteristic chemical shifts, which further confirms its successful synthesis (Figure S13 in the Supplementary Materials). Similarly, the structures of the copolymers TPP1–3 exhibit all the characteristic chemical shifts in the ^1^H- and ^13^C-NMR spectra (Figures S6–S8 and S14 in the Supplementary Materials).

**Figure 2:**
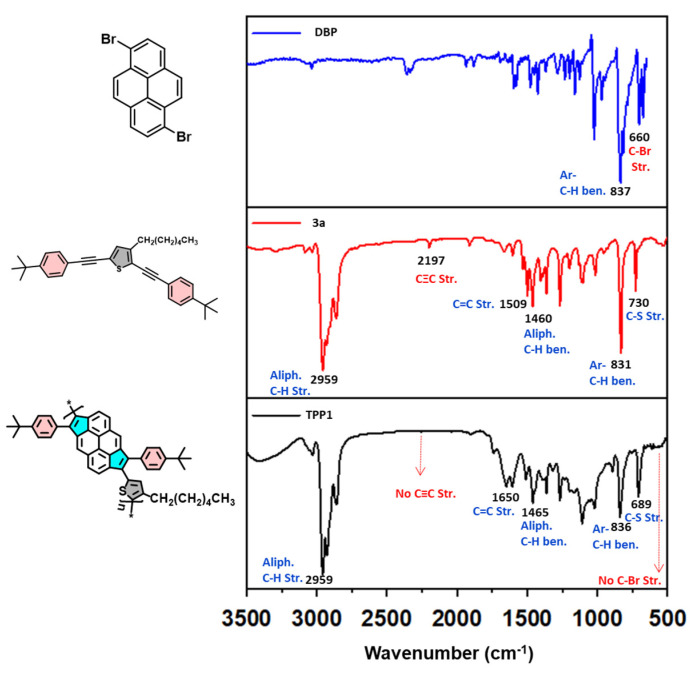
Comparative FT-IR absorption spectra of DBP, 3a, and TPP1.

The comparative FT-IR absorption spectra of DBP, 3a and TPP1 shown in Figure 2, provide a clear indication of the absence of two fingerprint bands observed in monomers DBP and 3a from the FT-IR absorption of TPP1, namely, the C-Br stretching vibration in DBP, which is observed at 660 cm^−1^ [44] and the alkynyl (C≡C) stretching vibration in 3a, which is detected at 2197 cm^−1^ [45], hence proving a complete cyclopentannulation reaction. In addition, the presence of all the characteristic peaks in the FT-IR spectrum of TPP1 confirms its formation, notably the aliphatic C-H and aromatic C=C stretching vibrations at 2959 cm^−1^ and 1650 cm^−1^, respectively, and the C-H bending vibrations of the aliphatic and aromatic groups at 1465 cm^−1^ and 836 cm^−1^, respectively [46,47], as well as the absorption band seen at 689 cm^−1^ and that is assigned to C-S stretching vibration of thiophene [48]. Similarly, all the other target copolymers disclose similar characteristic peaks, which confirm their successful synthesis (Figures S21 and S22 in the Supplementary Materials).

**Figure 3:**
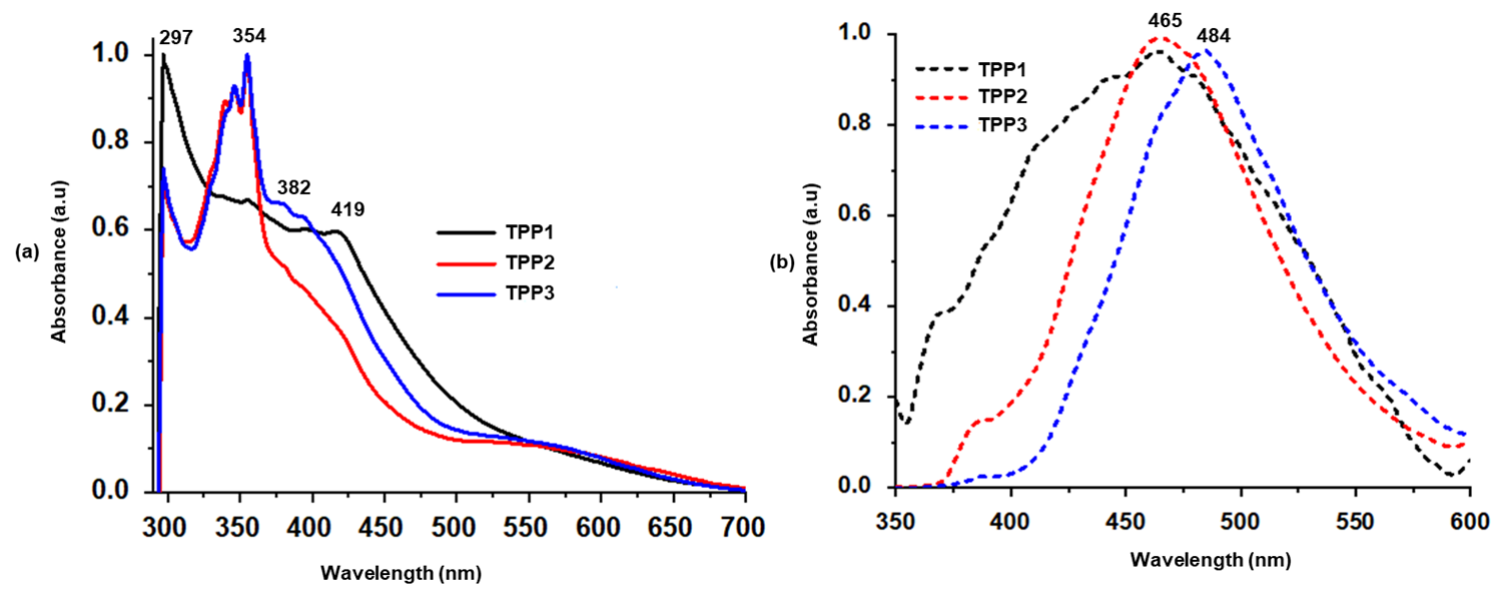
Normalized UV–Vis absorption (CM = 10^−8^ M in THF) (**a**) and emission (**b**) spectra (excited at 300 nm) of TPP1–3.

As shown in Figure 3 below, the UV–Vis absorption and fluorescence spectroscopies denote the photophysical properties of TPP1–3. The UV–Vis absorption spectrum of TPP1 shows a maximum absorption band at 297 nm in addition to a shoulder band detected at 419 nm. On the other hand, the UV–Vis absorption spectra of TPP2 and TPP3, where the former contains three fused thiophene rings while the latter bears a terthiophene derivative per each repeating unit, exhibit an absorption peak maximum at 354 nm. Interestingly, the emission spectra of TPP1–3 display broad emission bands with peak maxima at 465 nm and 484 nm for TPP1,2 and TPP3, respectively. It should be noted that TPP2,3 shows a low-energy absorption band at 500–650 nm but with a very low intensity.

The thermal stability of conjugated polymers TPP1–3 were investigated by the thermogravimetric analysis (TGA), revealing their 10% weight loss temperatures in the range of 390–413 °C, which indicates their relatively high thermal stability (Figure S28 in the Supplementary Materials). It is worthwhile to note that the relatively good solubility of TPP1 in common organic solvents has permitted the determination of its molecular weight by gel permeation chromatography (Figure S29 in the Supplementary Materials), which revealed a weight average molar mass Mw of ~12.3 KDa and number average molar mass Mn of ~6.2 KDa, thus showing a polydispersity index (Ð = M_w_/M_n_) of 1.98.

## 4. Surface Area and Porosity Analysis

Nitrogen adsorption experiments at 77 K and low relative pressure revealed both the surface areas and porous properties of conjugated copolymers TPP1–3 (Figure S30 in the Supplementary Materials). Brunauer–Emmett–Teller (BET) surface areas and pore volumes were obtained from the nitrogen sorption isotherms, disclosing a surface area of ~67 m^2^ g^−1^ and a pore volume of 0.091 cm^3^ g^−1^ for the conjugated copolymer TPP1. On the other hand, copolymers TPP2 and TPP3 exhibit relatively lower BET surface areas than the former, with 36 m^2^ g^−1^ and 32 m^2^ g^−1^, respectively, and pore volumes of 0.082 cm^3^ g^−1^ and 0.035 cm^3^ g^−1^, respectively. The low BET surface areas of TPP1–3 can be attributed to the planar and conjugated structure of the dicyclopenta[cd,jk]pyrenyl units in the copolymers’ backbones whose supramolecular π-π stacking is believed to hinder the formation of large pockets needed to obtain a large surface area [49].

## 5. Iodine Uptake

**Figure 4:**
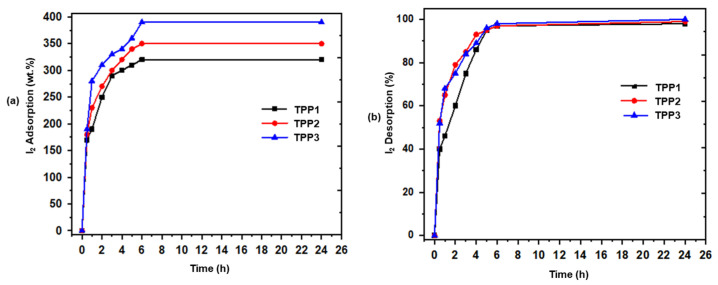
Wt.% of iodine adsorption (**a**) and % of iodine desorption (**b**) graphs of TPP1–3.

Iodine uptake properties of TPP1–3 were examined using gravimetric analysis, where the amount of iodine adsorbed by the copolymers was measured by taking a 10 mg sample of the latter in an open glass flask and kept inside a sealed glass vessel that contained excess solid iodine at 80 °C and under atmospheric pressure. The quantity of adsorbed iodine was recorded at different time intervals until it attained equilibrium (Figure 4). Interestingly, the wt.% of I_2_ adsorbed by the conjugated copolymers was found to be in the range of 320–390 wt.% (Table 1), where the maximum iodine adsorption capacities for copolymers TPP1 and TPP2 were found to be 320 wt.% and 350 wt.%., respectively, after 24 h of exposure to iodine fumes (Table 1 and Figure 4). Interestingly, TPP3, i.e., the copolymer that bears terthiophene rings, exhibited the highest iodine uptake, reaching a maximum of 390 wt.%. in 24 h (Table 1 and Figure 4), which promotes it as a promising candidate given several advantages. Namely, its simple synthesis and purification, especially when compared with several adsorbents reported in the literature, which require complex synthetic and/or isolation steps and whose iodine adsorption values are lower than that of TPP3 [50,51,52,53,54,55].

**Figure 5:**
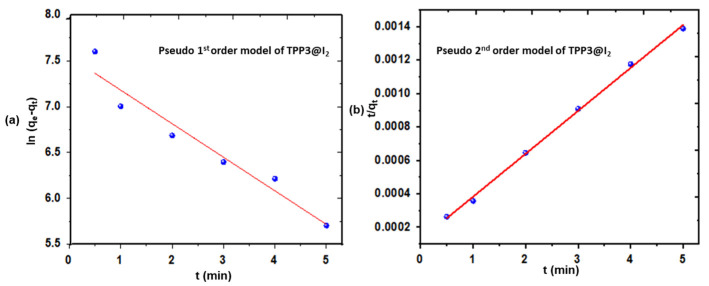
Pseudo-first-order (**a**) and pseudo-second-order (**b**) models of TPP3@I_2._

The adsorption mechanism of iodine by TPP1–3 was investigated by carrying out kinetic experiments using pseudo-first-order and pseudo-second-order kinetic models, as expressed by Equations (2) and (3) shown in the experimental part. Figure 5 portrays the calculated adsorption capacity at equilibrium, q_e,cal_, from the pseudo-first-order model, which was obtained by plotting ln(q_e_–q_t_) vs. t, whereas the plot of t/q_t_ vs. t was used to determine q_e,cal_ for the pseudo-second-order model. It is interesting to note that the data compiled for both models and shown in Table 2 reveal that the correlation coefficient, R^2^, derived from the linear correlation of the pseudo-second-order model (R^2^ = 0.9976) is higher than the one obtained from the pseudo-first-order model (R^2^ = 0.9278). Furthermore, Table 2 divulges that the ~3900 mg g^−1^ experimental iodine adsorption capacity at equilibrium, q_e,exp_, is in better agreement with the calculated iodine adsorption capacity, q_e,cal_, derived from the pseudo-second-order model, which was found to be ~3887 mg g^−1^, when compared with that determined from the pseudo-first-order model, and which portrays a value of ~1894 mg g^−1^. This strongly suggests that the adsorption of iodine by TPP3 follows the pseudo-second-order kinetic model. Similarly, TPP1,2 also follows pseudo-second-order kinetic models (Figures S31 and S32 in the Supplementary Materials).

**Figure 6:**
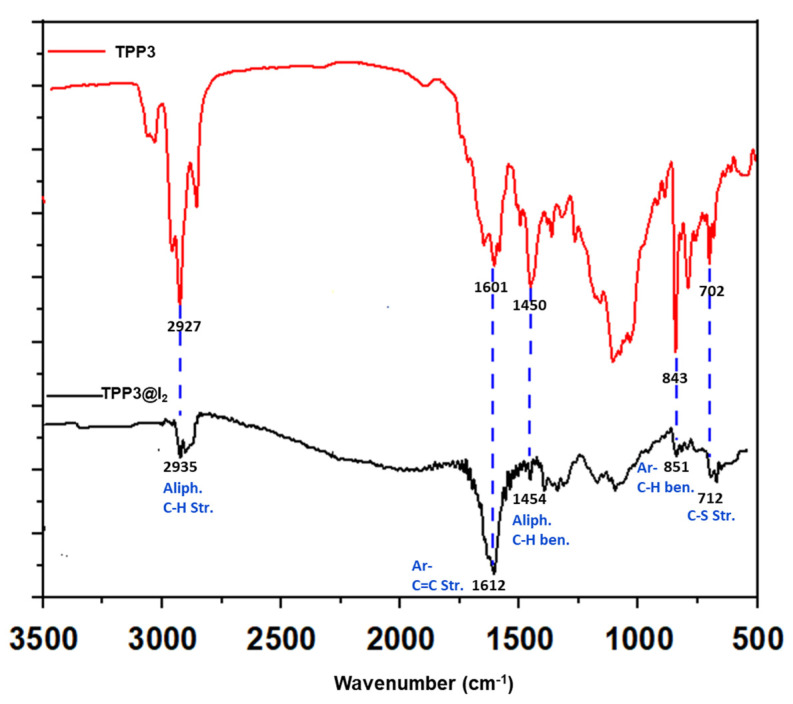
Comparative FTIR spectrum of TPP3 (**up**) and TPP3@I_2_ (**down**).

Iodine adsorption studies by TPP1–3 using FTIR spectroscopy depict several variations in the vibration bands of the hitherto mentioned conjugated copolymers loaded with iodine (i.e., TPP1–3@I_2_) when compared with their pristine polymers. Figure 6 displays the comparative FTIR spectrum of TPP3 before and after iodine capture: TPP3@I_2_ clearly reveals major shifts caused by iodine adsorption in the aliphatic C-H stretching and bending bands as well as aromatic C=C stretching and C-H bending besides C-S stretching vibrations bands. These conspicuous changes in the FTIR spectrum of TPP3@I_2_ prove the interaction between the electron-rich parts of TPP3 with iodine species [56]. In addition, the thiophene groups, which bear sulfur heteroatoms in the conjugated copolymer backbone, are believed to play a pivotal role in iodine uptake by acting as additional binding sites for the latter [57].

**Figure 7:**
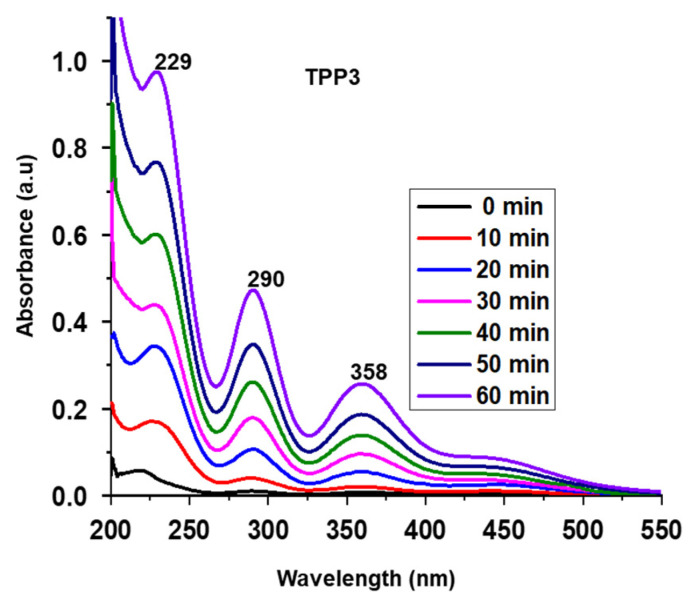
UV–Vis absorption spectra upon immersion of TPP3@I_2_ in ethanol.

Desorption experiments were carried out where the iodine-containing conjugated copolymers TPP1–3@I_2_ were heated in air at 120 °C (Figure 4 and Table 1), releasing almost all the adsorbed iodine (~97%) within the first 6 h, followed by quantitative desorption after 24 h of heating TPP1–3@I_2_ samples. Iodine release was further analyzed by immersing TPP1–3@I_2_ in ethanol because the latter is renowned as a very good solvent of iodine, thus causing the diffusion of the halogen from TPP1–3@I_2_ into ethyl alcohol solution as proven by UV–Vis spectra recorded at different time intervals (Figure 7 and Figures S34–S36 in the Supplementary Materials) and which disclosed the increase in the absorbance intensity maxima that correspond to iodine, namely at ~229 nm (typical for I_2_) with two additional peaks at ~290 nm and ~358 nm (specific to polyiodide ions), hence further confirming the halogen desorption from TPP1–3@I_2_ under ambient conditions [58]. After 60 min of soaking the TPP1–3@I_2_ sample in ethanol, the intensity of absorption did not vary, thus implying reaching equilibrium. These experimental observations confirm that copolymers TPP1–3 can be easily regenerated either by simple heating or immersion in ethanol, which promotes them as renewable materials.

**Figure 8:**
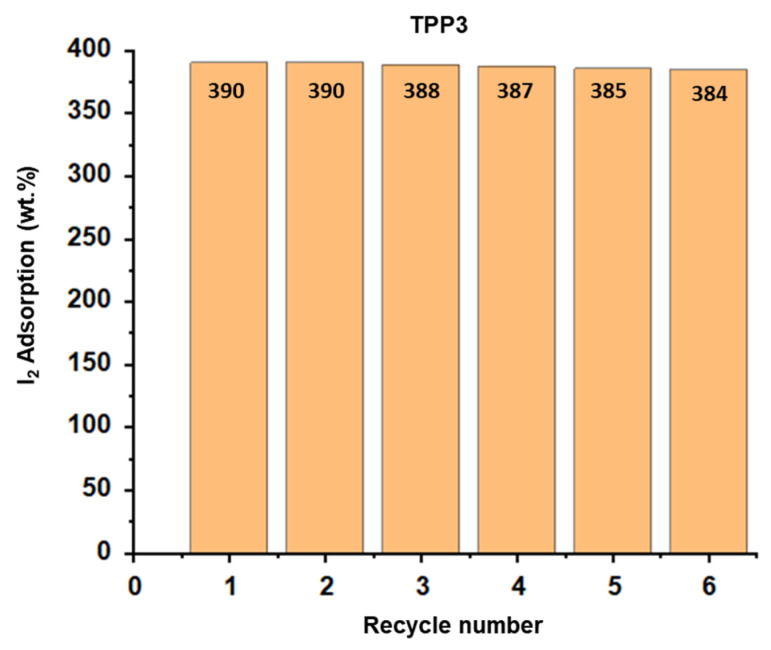
Regeneration performance of iodine adsorption by TPP3.

Recyclability tests of the iodine-adsorbed conjugated copolymers were performed using TPP3 as a model. Thus, TPP3@I_2_ was heated at 120 °C for 24 h to ensure the complete release of the adsorbed iodine from the copolymer, followed by exposing the regenerated sample TPP3(R) to iodine vapors whose uptake values were measured gravimetrically using the procedure described above. The aforementioned regeneration procedure was repeated using TPP3(R), which revealed sustaining an excellent uptake capacity even after six successive adsorption-desorption cycles, showing only a slight 1–6% decrease in its iodine uptake efficiency throughout the whole experiment (Figure 8).

## 6. Nickel Ion Adsorption

**Figure 9:**
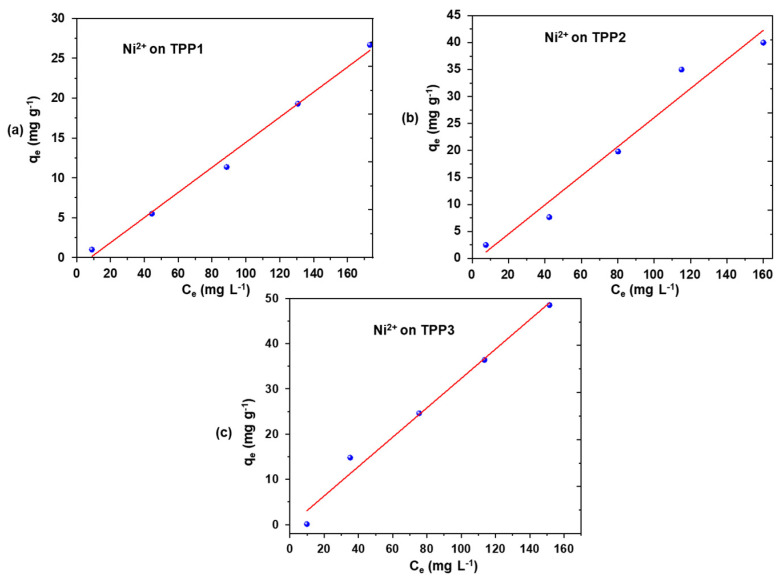
The plot of q_e_ (mg g^−1^) Vs. C_e_ (mg L^−1^) for the adsorption of Ni^2+^ by TPP1–3. (**a**) TPP1 (**b**) TPP2 and (**c**) TPP3.

Nickel ions adsorption from aqueous solutions by TPP1–3 was investigated by immersing an aliquot of the latter in standard solutions of Ni^2+^ ranging from 10 to 200 mg L^−1^. The concentration of Ni^2+^ in solution before and after the adsorption by TPP1–3 was measured using UV–Vis absorption spectrophotometry, whereas the Ni^2+^ equilibrium adsorption capacity q_e_ (mg g^−1^) was calculated using Equation (4), shown in the experimental part. Amongst all the three cyclopentannulated copolymers TPP1–3, the one bearing terthiophene units, i.e., TPP3, displayed the highest equilibrium adsorption capacity qe (mg g^−1^) for Ni^2+^ from the aqueous solution, reaching up to 48.5 mg g^−1^, which is promising, especially when compared with several values that have been reported in the literature [59,60,61,62,63]. On the other hand, conjugated copolymers TPP1 and TPP2 portray nickel ions’ equilibrium adsorption capacities, q_e_ (mg g^−1^), of 27 mg g^−1^ and 40 mg g^−1^, respectively (Figure 9).

## 7. Conclusions

New conjugated copolymers TPP1–3 bearing alternating units of dicyclopenta[cd,jk]pyrenyl groups and various thiophene derivatives were synthesized in excellent yields using a convenient one-step palladium-catalyzed [3+2] cyclocondensation polymerization reaction. Iodine uptake analysis of TPP1–3 demonstrated sizeable adsorption capacities up to 390 wt.% (q_e_ = 3900 mg g^−1^) with an adsorption mechanism that follows a pseudo-second-order kinetic model. Regeneration tests showcased the high efficiency of TPP3 even after six successive iodine adsorption-desorption cycles. Furthermore, the target copolymers exhibited very good capture properties of nickel ions from aqueous solutions with a promising equilibrium adsorption capacity (q_e_) for TPP3 of 48.5 mg g^−1^. The conjugated copolymers presented herein reveal several advantages, such as their versatile one-pot efficient polymerization reaction as well as excellent chemical and thermal stabilities. On top of that, TPP1–3 discloses high uptake values of iodine and nickel ions, thus promoting them as promising adsorbent materials for wastewater purification and environmental remediation.

## Figures and Tables

**Table 1:** Summary of iodine adsorption by copolymers TPP1–3.

Entry	Polymer	Wt.% I_2_ Adsorption after 24 h	% I_2_ Desorption after 24 h
1	TPP1	320	98
2	TPP2	350	99
3	TPP3	390	100

**Table 2:** Pseudo-first-order and pseudo-second-order model parameters for TPP3@I_2._

Polymer		Pseudo 1st-Order Model			Pseudo 2nd-Order Model		
	q_e,exp_ (mg g^−1^)	q_e,cal_ (mg g^−1^)	k_1_ (min^−1^)	R^2^	q_e,cal_ (mg g^−1^)	k_2_ (min^−1^)	R^2^
TPP3@I_2_	3900	1894	−0.01526	0.9278	3887	0.000528	0.9976

## Data Availability

The raw/processed data required to reproduce these findings can be shared upon demand.

## Supplementary Materials

The following supporting information can be downloaded at: https://www.mdpi.com/article/10.3390/polym15204153/s1, Figure S1–S8: ^1^H NMR spectrum of 3a-d, TPM, and TPP1–3; Figure S9–S14: ^13^C NMR spectrum of 3a-d, TPM, and TPP1; Figure S15–S22: FTIR spectrum of 3a–d, TPM, and TPP1–3; Figure S23–S27: EI–MS spectrum of 3a–d and TPM; Figure S28: TGA thermograms of TPP1–3; Figure S29: Normalized GPC chromatogram of TPP1; Figure S30: Nitrogen adsorption and desorption isotherms of TPP1–3; Figure S31–S33: CO_2_ adsorption and desorption isotherms of TPP1–3; Figure S34–S36: Kinetic studies of TPP1–3@I_2_; Figure S37–S39: UV–Vis absorption spectra of TPP1@I_2_ in ethanol.
