# Supplementary material for: Iodine and Nickel Ions Adsorption by Conjugated Copolymers Bearing Repeating Units of Dicyclopentapyrenyl and Various Thiophene Derivatives
Source: Polymers (Basel). 2023 Oct 19;15(20):4153. doi: 10.3390/polym15204153 (PMC10611155; doi:10.3390/polym15204153)
Supplement: Supplementary file 1 [file polymers-15-04153-s001.zip › polymers-2673628-supplementary.pdf]

Supporting Information

Iodine and Nickel Ions Adsorption by Conjugated Copolymers Bearing Repeating Units of Dicyclopentapyrenyl and Various Thiophene Derivatives

Suchetha Shetty,[1,2] Noorullah Baig,[1,2] Sk Abdul Wahed,[3] Atikur Hassan,[3] Neeladri Das [3] and Bassam Alameddine [1,2,*]

[1] Department of Mathematics and Natural Sciences, Gulf University for Science and Technology, Kuwait.

[2] Functional Materials Group, Gulf University for Science and Technology, Kuwait.

[3] Department of Chemistry, Indian Institute of Technology Patna, Patna, 801106, Bihar, India; atikurhassan@gmail.com (A.H.); neeladri@iitp.ac.in (N.D.)

Tel: +965 2530 7111.

E-mail address: alameddine.b@gust.edu.kw

Contents

Synthesis of **3d**	(i)
CO_2 gas uptake studies	(ii)
^{1}H NMR spectrum of **3a-d**, **TPM**, **TPP1-3**	Figures S1-S8
^{13}C NMR spectrum of **3a-d**, **TPM**, **TPP1**	Figures S9-S14
FTIR spectrum of **3a-d**, **TPM**, **TPP1-3**	Figures S15-S22
EI-MS spectrum of **3a-d** and **TPM**	Figures S23-S27
TGA thermograms of **TPP1-3**	Figure S28
Normalized GPC chromatogram of **TPP1**	Figure S29
Nitrogen adsorption and desorption isotherms of **TPP1-3**	Figure S30
Kinetic studies of **TPP1-3@I_2**	Figures S31-S33
UV-Vis absorption spectra of **TPP1@I_2** in ethanol	Figures S34-S36

*(i). Synthesis of 2-((4-(tert-butyl)phenyl)ethynyl)-5-hexylthiophene (**3d**)*

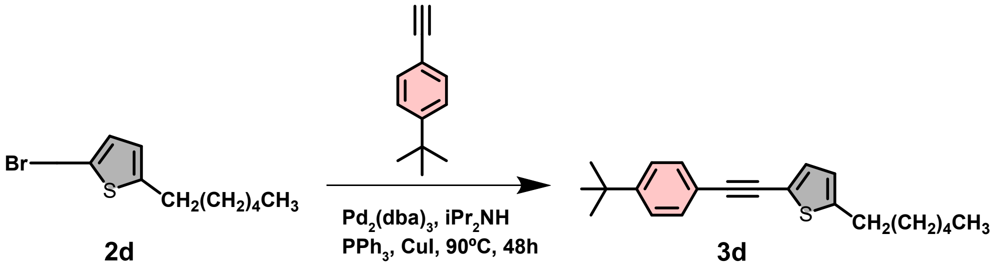

3d was prepared following procedure A with 2-bromo-5-hexylthiophene **2d** (0.3 g, 1.2 mmol, 1 eq.), 1-(tert-butyl)-4-ethynylbenzene 1a (0.46 g, 2.9 mmol, 2.4 eq.) $Pd_2(dba)_3$ (6.7 mg, 0.0073 mmol, 0.6 mol%), PPh_3 (28 mg, 0.1 mmol), and CuI (4.6 mg, 0.02 mmol) in 10 mL of degassed diisopropylamine (iPr_2NH) at 90ºC for 2 days. pale pink solid (140 mg, 93%).^{1}H-NMR (600 MHz, CD_2Cl_2, ppm): δ 7.76 (d, 2H, J = 7.8 Hz, ArH), 7.66 (s, 2H, ArH), 7.56 (dd, 2H, J = 7.8, 6.6 Hz, ArH) 7.52 (m, 4H, J = 3.6 Hz, ArH) 7.45 (m, 4H, ArH), 1.55 (s, 6H, -CH3),1.37 (s, 18H, -CH3). 13C-NMR (150 MHz, CD2Cl2, ppm): δ 154.66, 152.35, 139.20, 131.80, 131.30, 126.50, 126.09, 122.94, 120.84, 90.44, 89.98, 47.47, 35.29, 31.50, 27.32. EI-HRMS: m/z calculated for $M^{\bullet+}$ C39H38 506.2974 found 506.2975.

(ii). CO_2 gas uptake studies

The CO_2 gas adsorption properties of **TPP1-3** were investigated by recording their CO_2 sorption isotherms with a pressure up to one atmosphere at temperatures of 273 K and 298 K (Figures S31-S33). The CO_2 uptake capacity of **TPP1** is 12.72 mg g^{-1} and 17.86 mg g^{-1} at 273 k and 298 K respectively. Furthermore, the conjugated polymers **TPP2** and **TPP3** display CO_2 uptake efficiency up to 17.53 mg g^{-1} and 18.20 mg g^{-1} at 273 K and 16.99 mg g^{-1} and 18.82 mg g^{-1} at 298 K, respectively

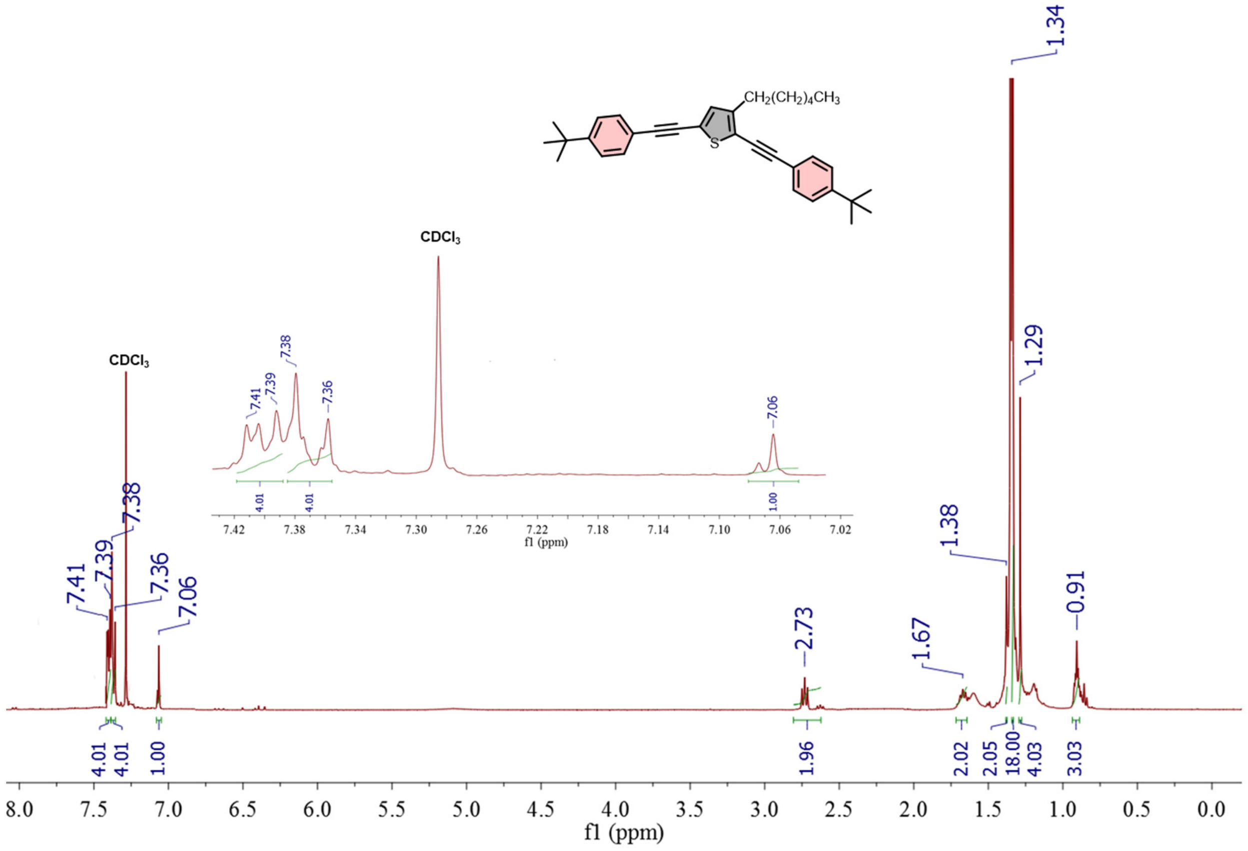

Figure S1 ^{1}H NMR spectrum of **3a**

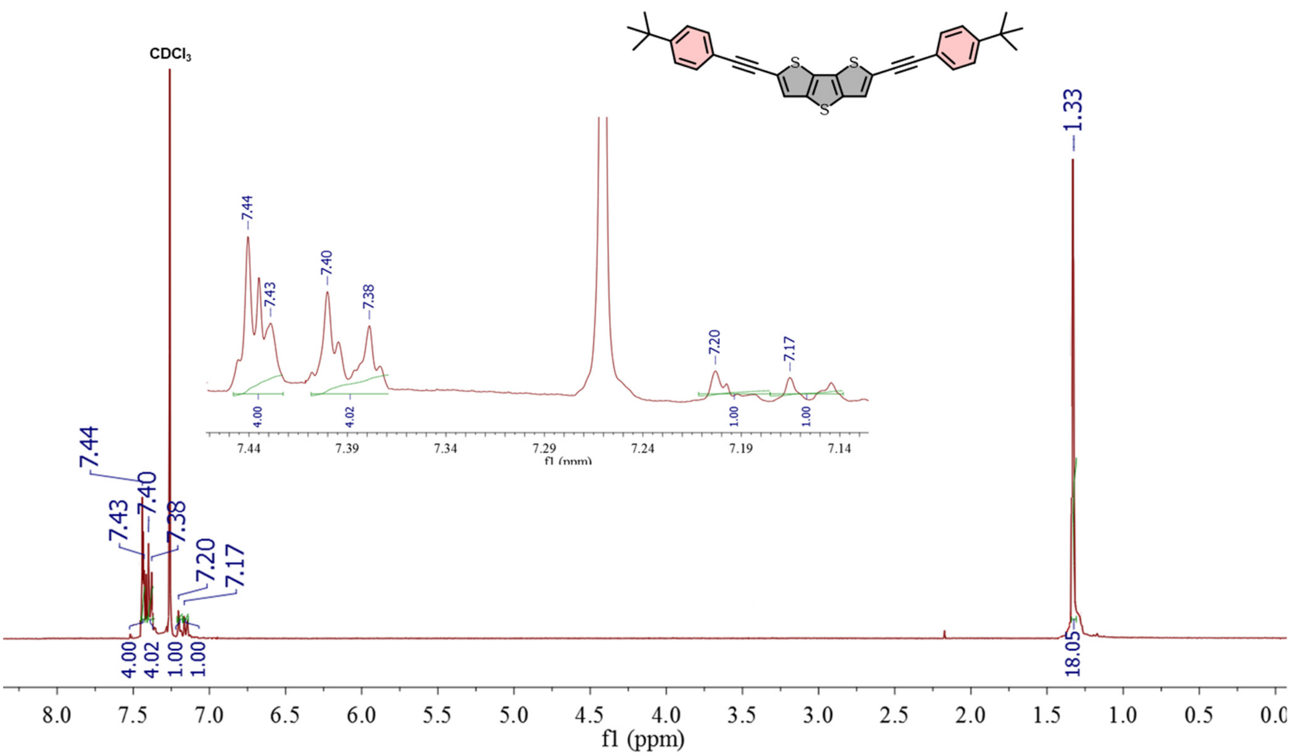

Figure S2 ^{1}H NMR spectrum of **3b**

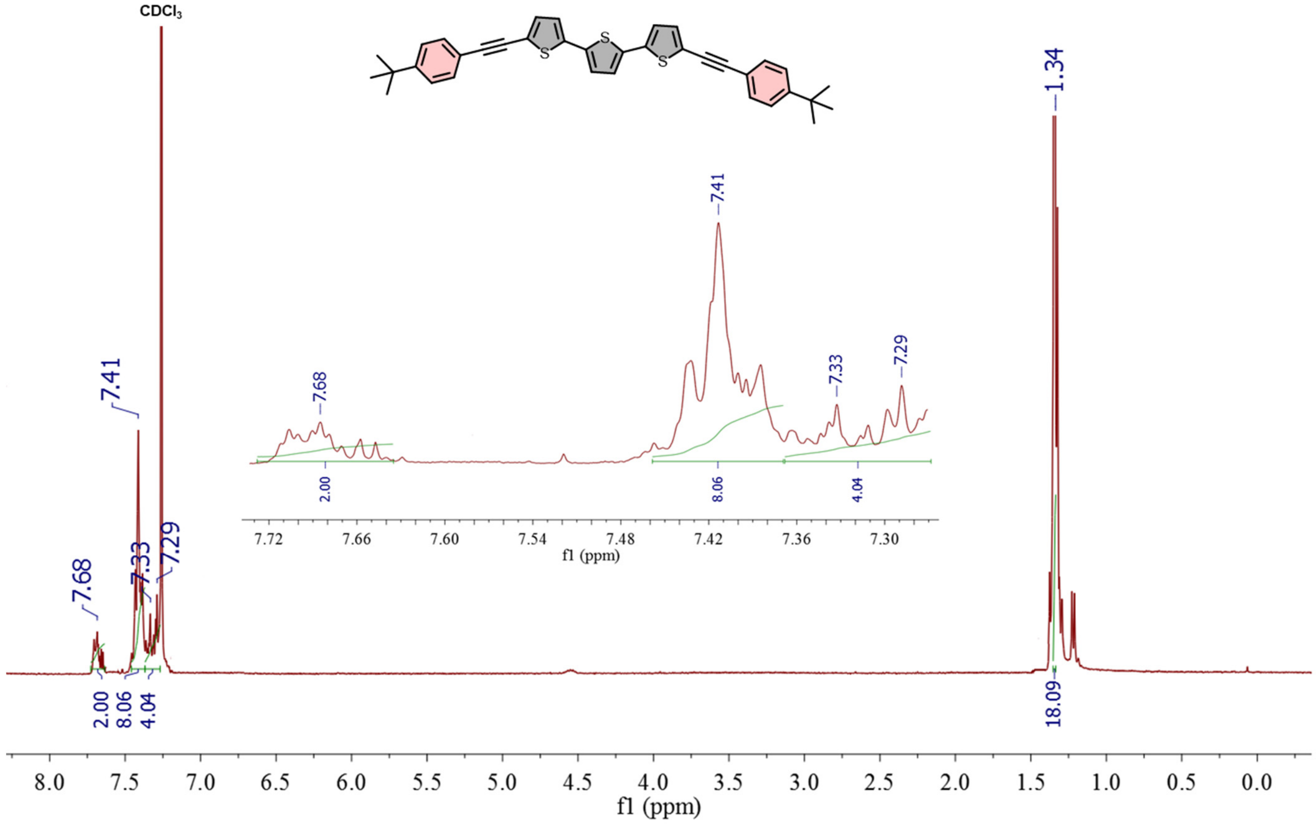

Figure S3 ^{1}H NMR spectrum of **3c**

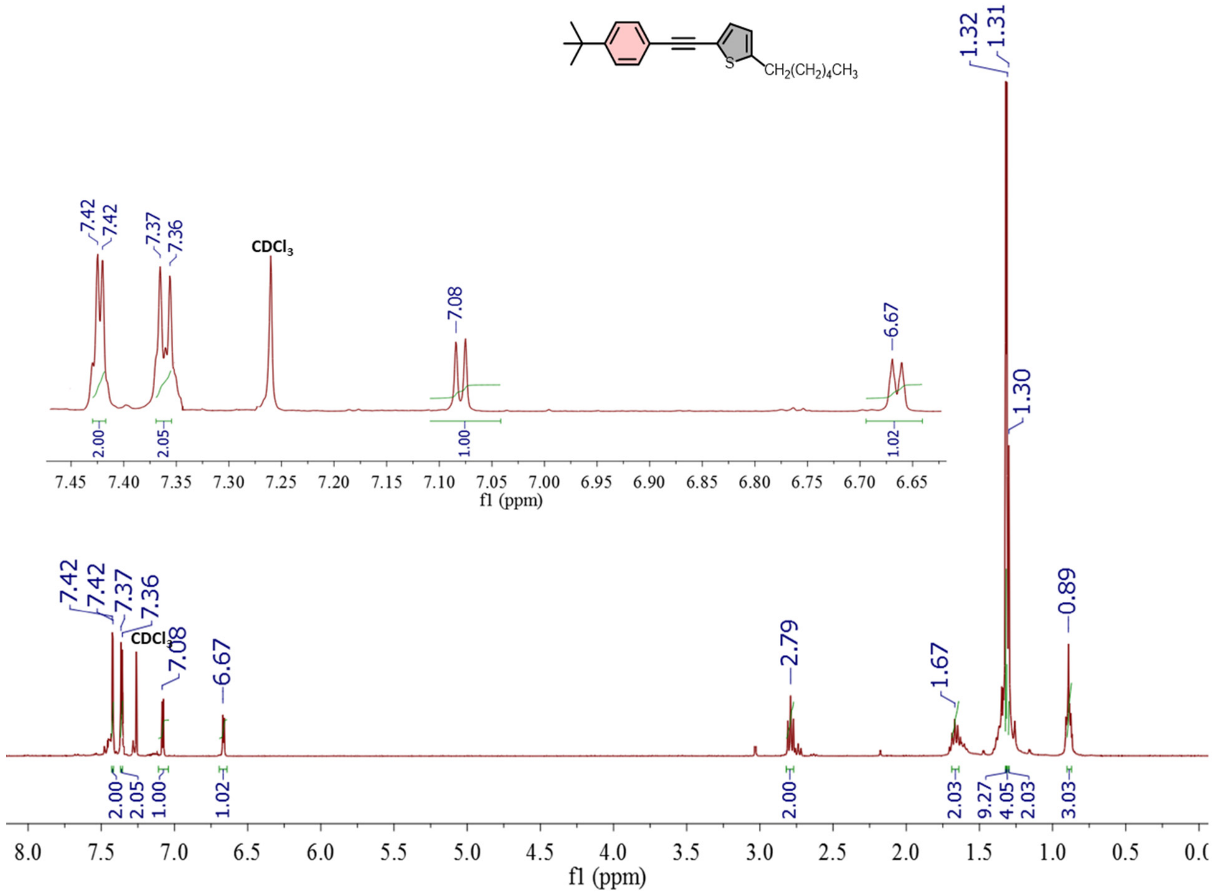

Figure S4 ^{1}H NMR spectrum of **3d**

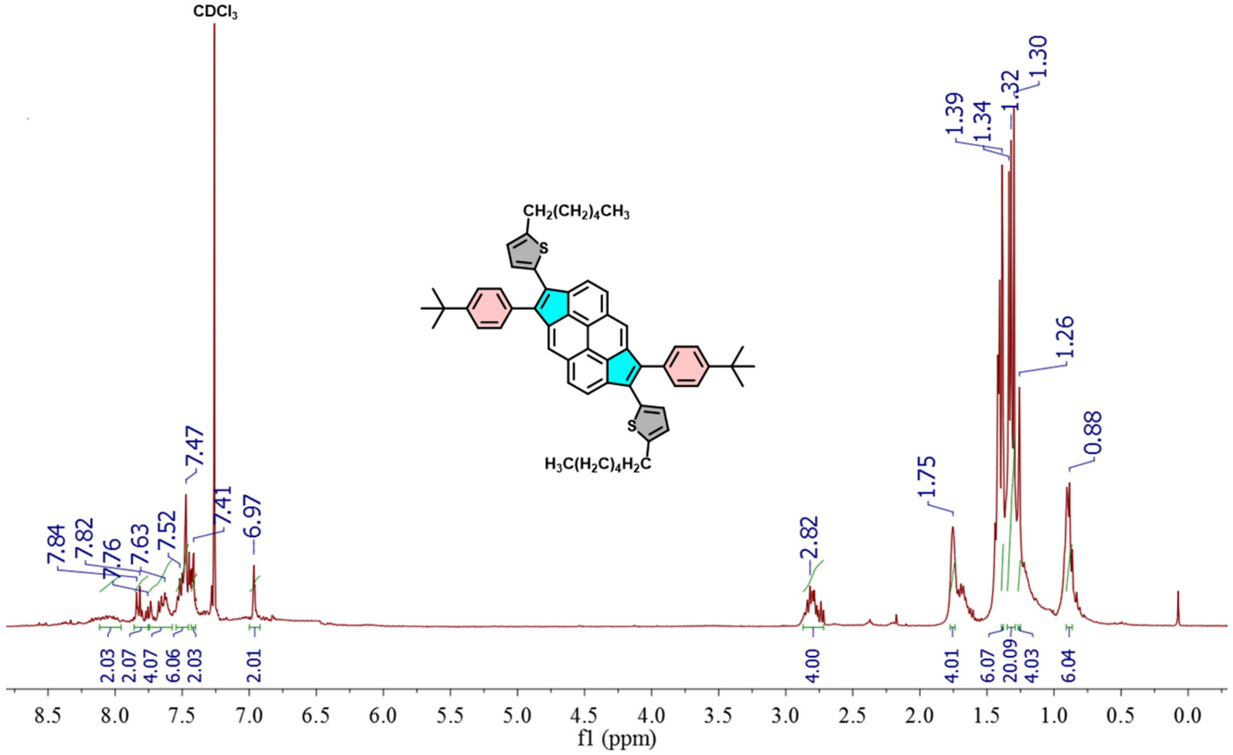

Figure S5 ^{1}H NMR spectrum of **TPM**

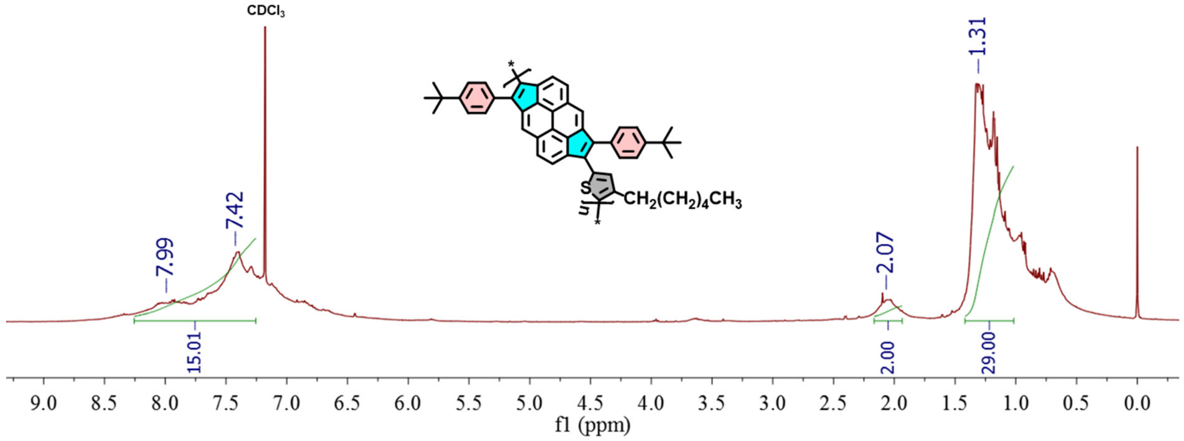

Figure S6 ^{1}H NMR spectrum of **TPP1**

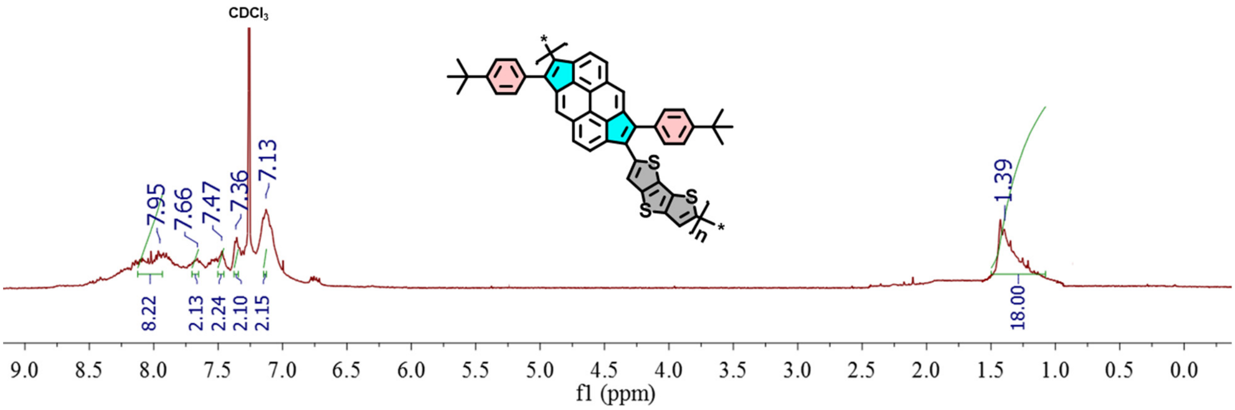

Figure S7 ^{1}H NMR spectrum of **TPP2**

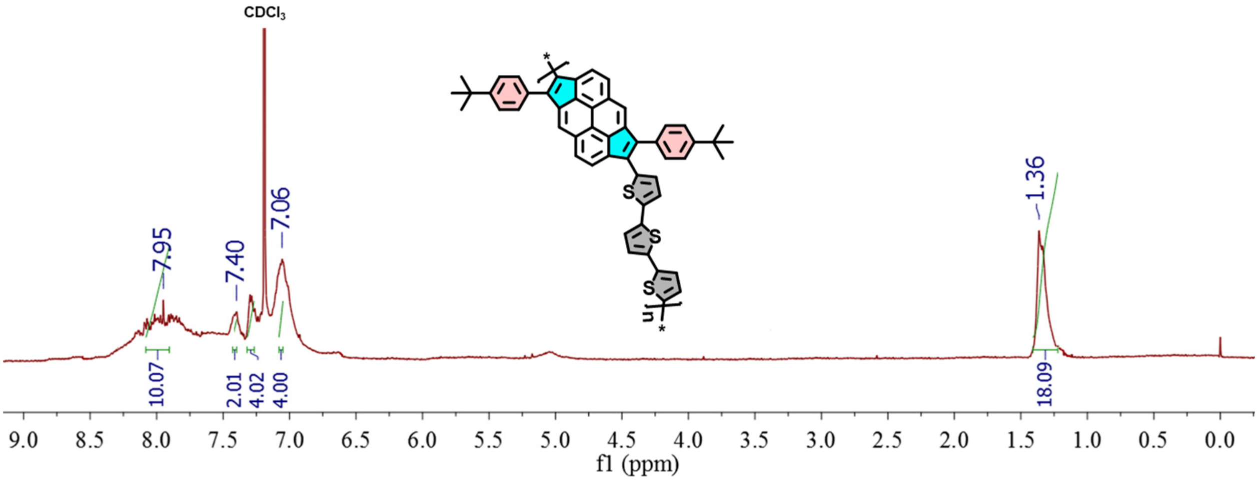

Figure S8 ^{1}H NMR spectrum of **TPP3**

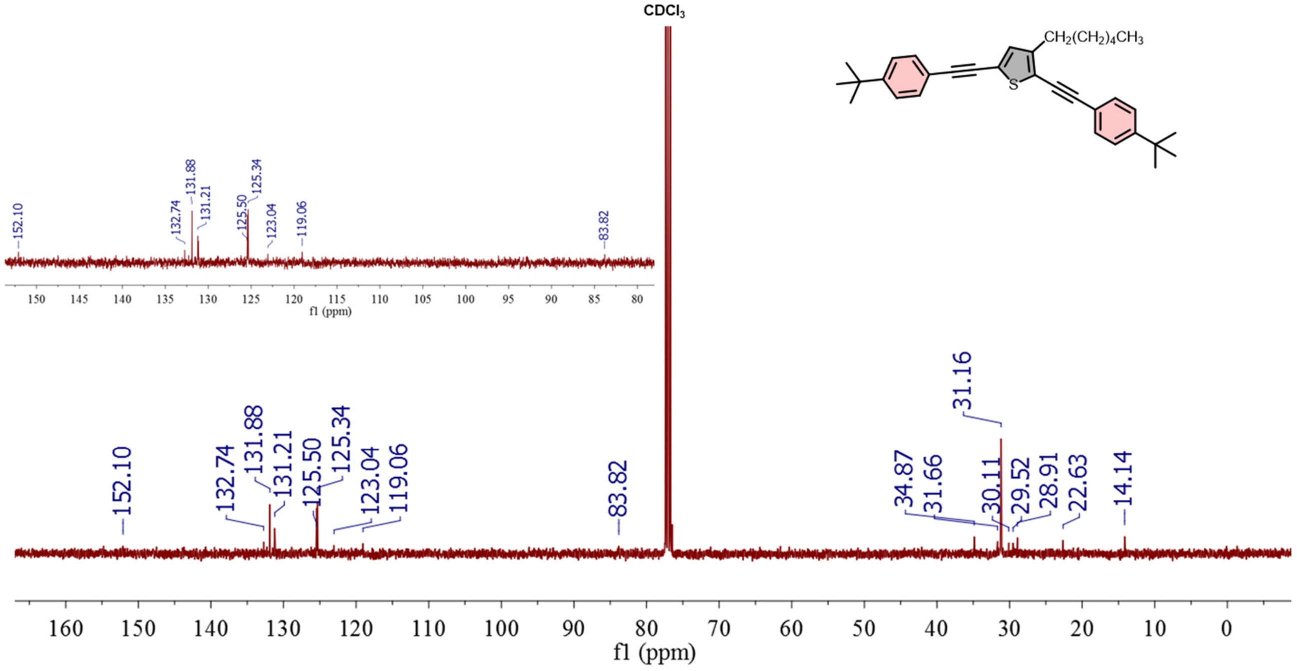

Figure S9 ^{13}C NMR spectrum of **3a**

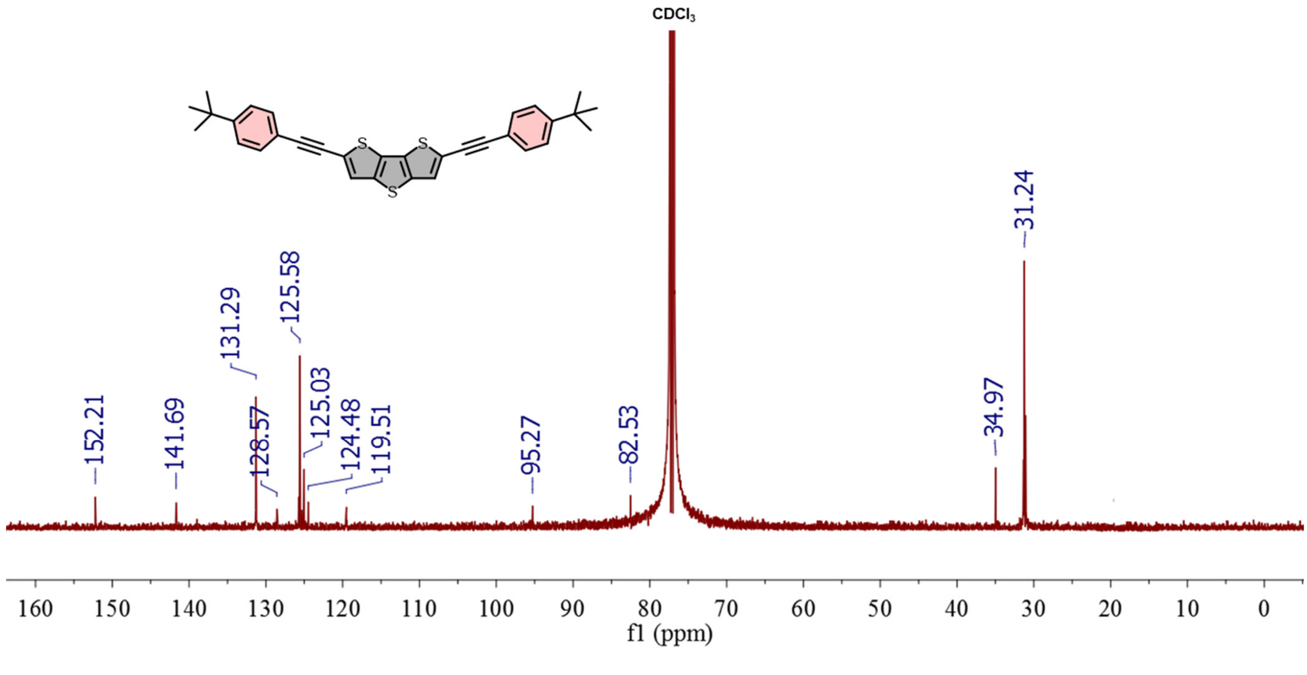

Figure S10 $^{13}CNMR$ spectrum of **3b**

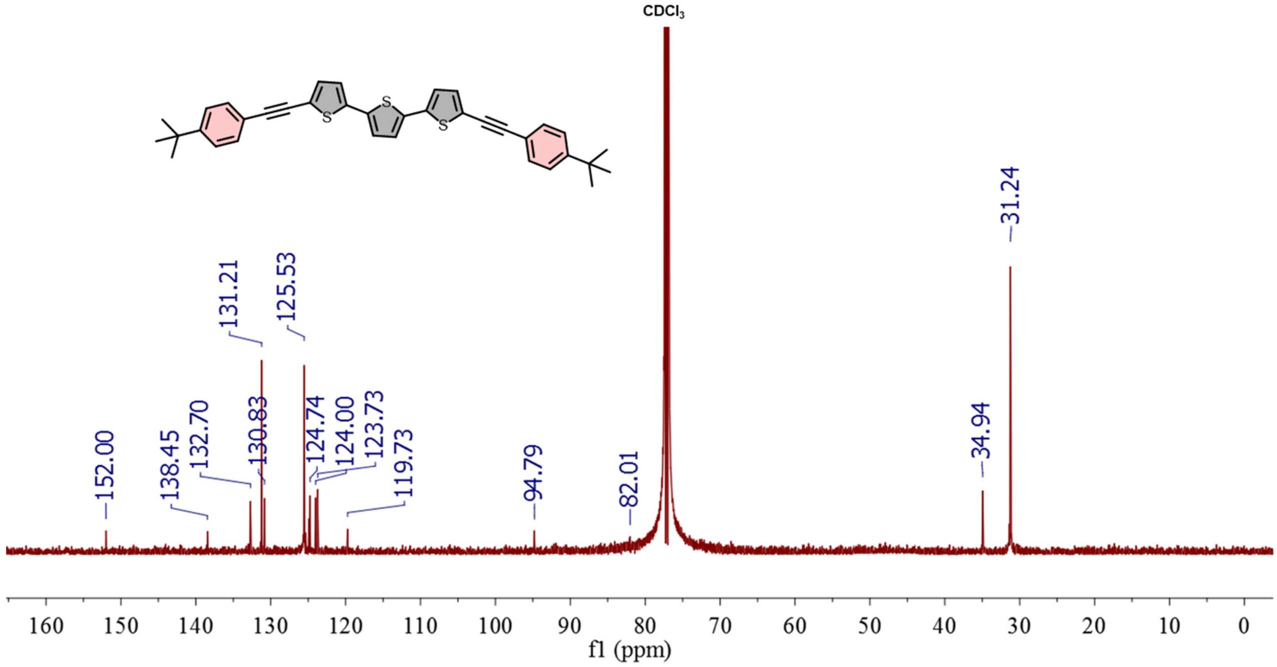

Figure S11 ^{13}C NMR spectrum of **3c**

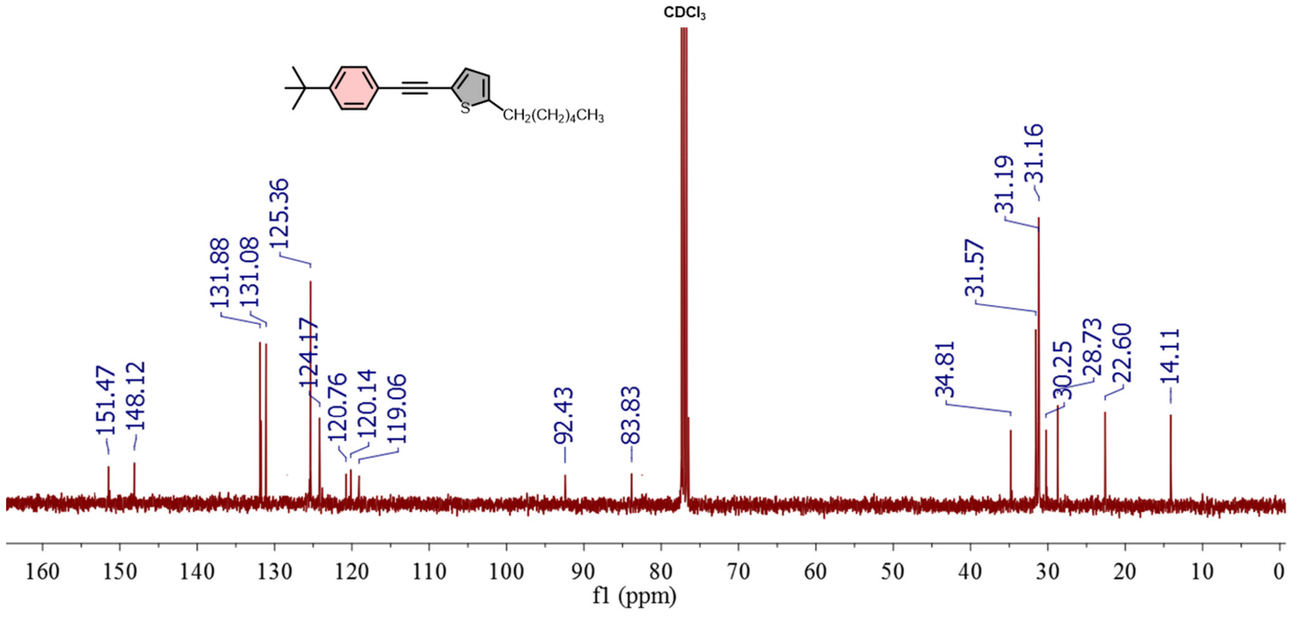

Figure S12 ^{13}C NMR spectrum of **3d**

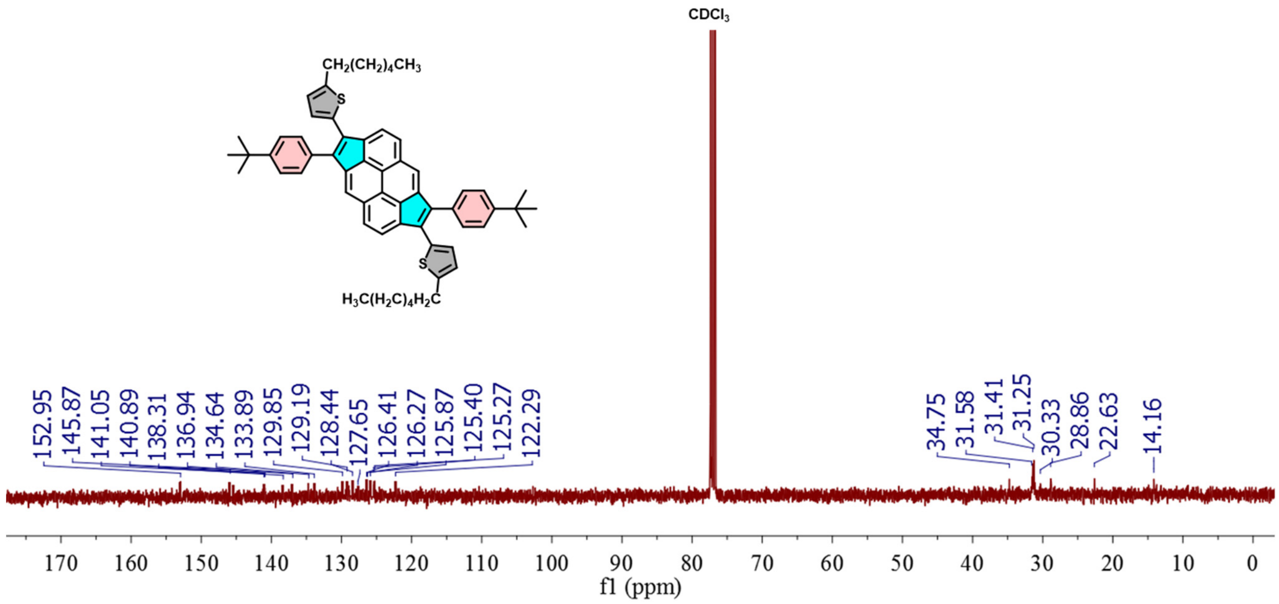

Figure S13 ^{13}C NMR spectrum of **TPM**

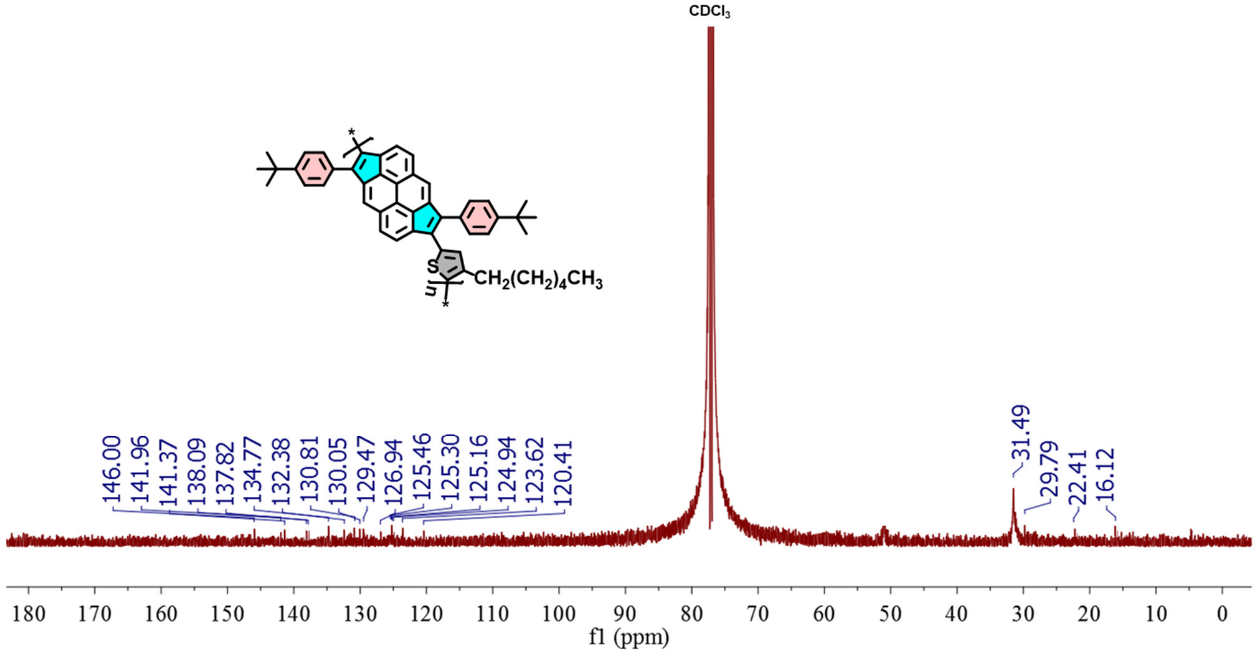

Figure S14 ^{13}C NMR spectrum of **TPP1**

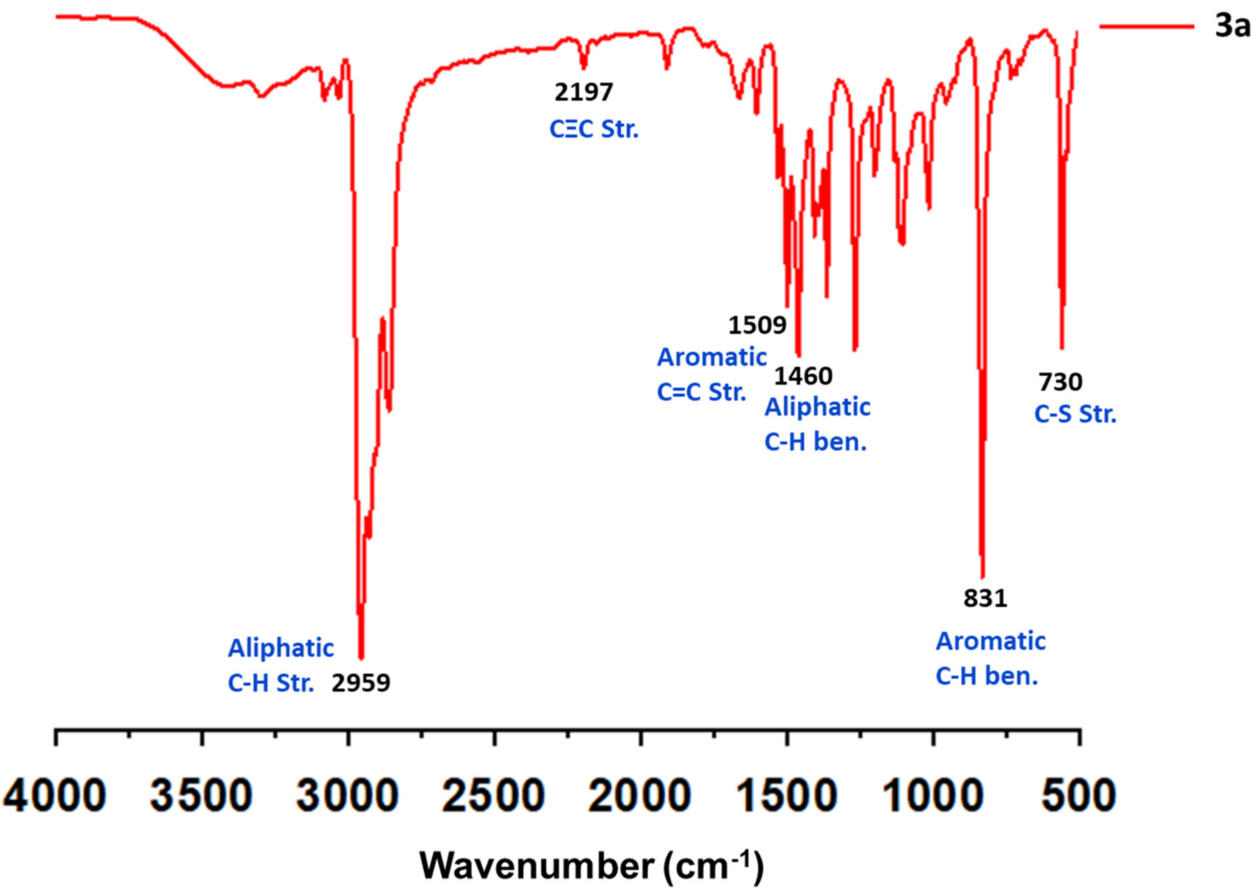

Figure S15 FTIR spectrum of **3a**

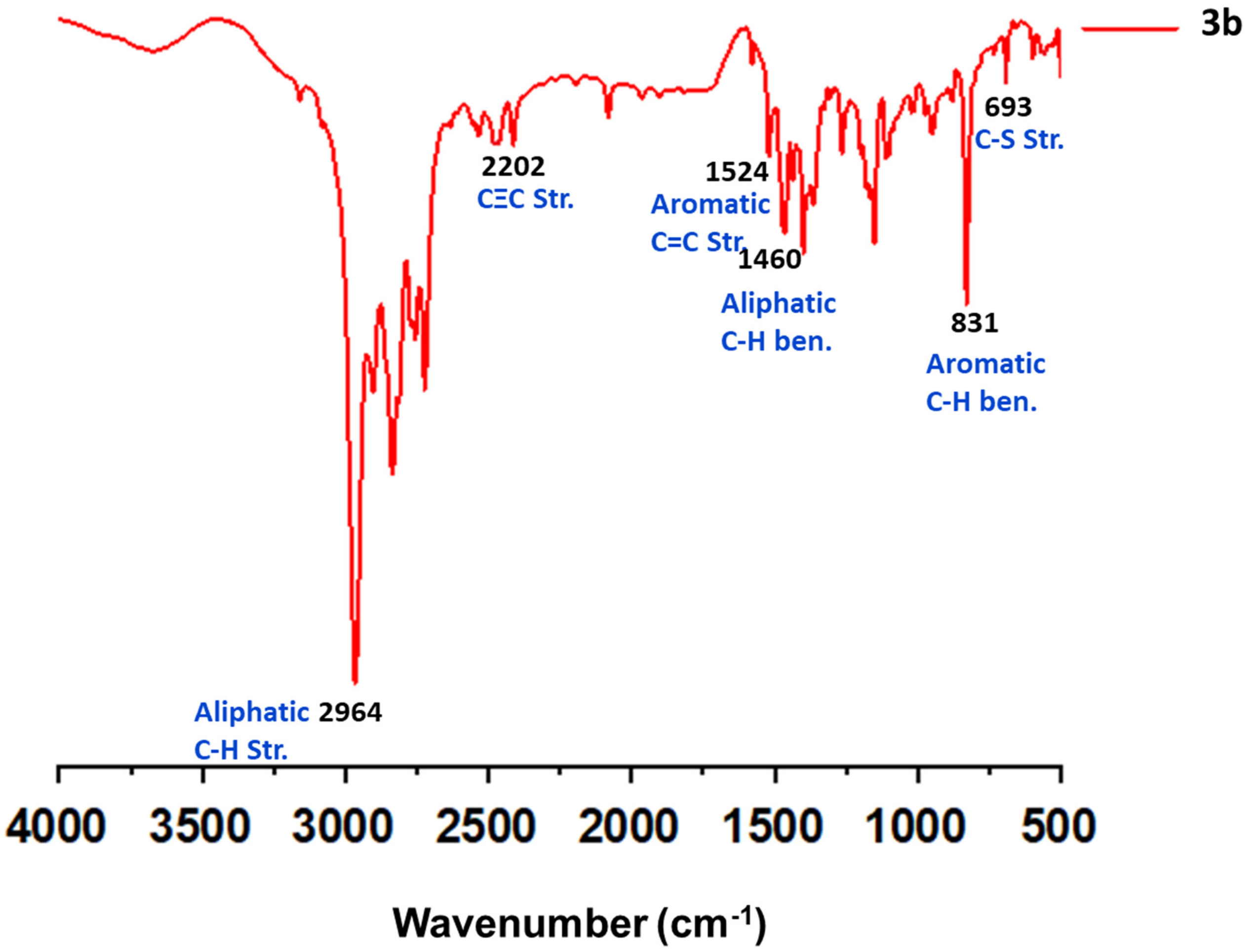

Figure S16 FTIR spectrum of **3b**

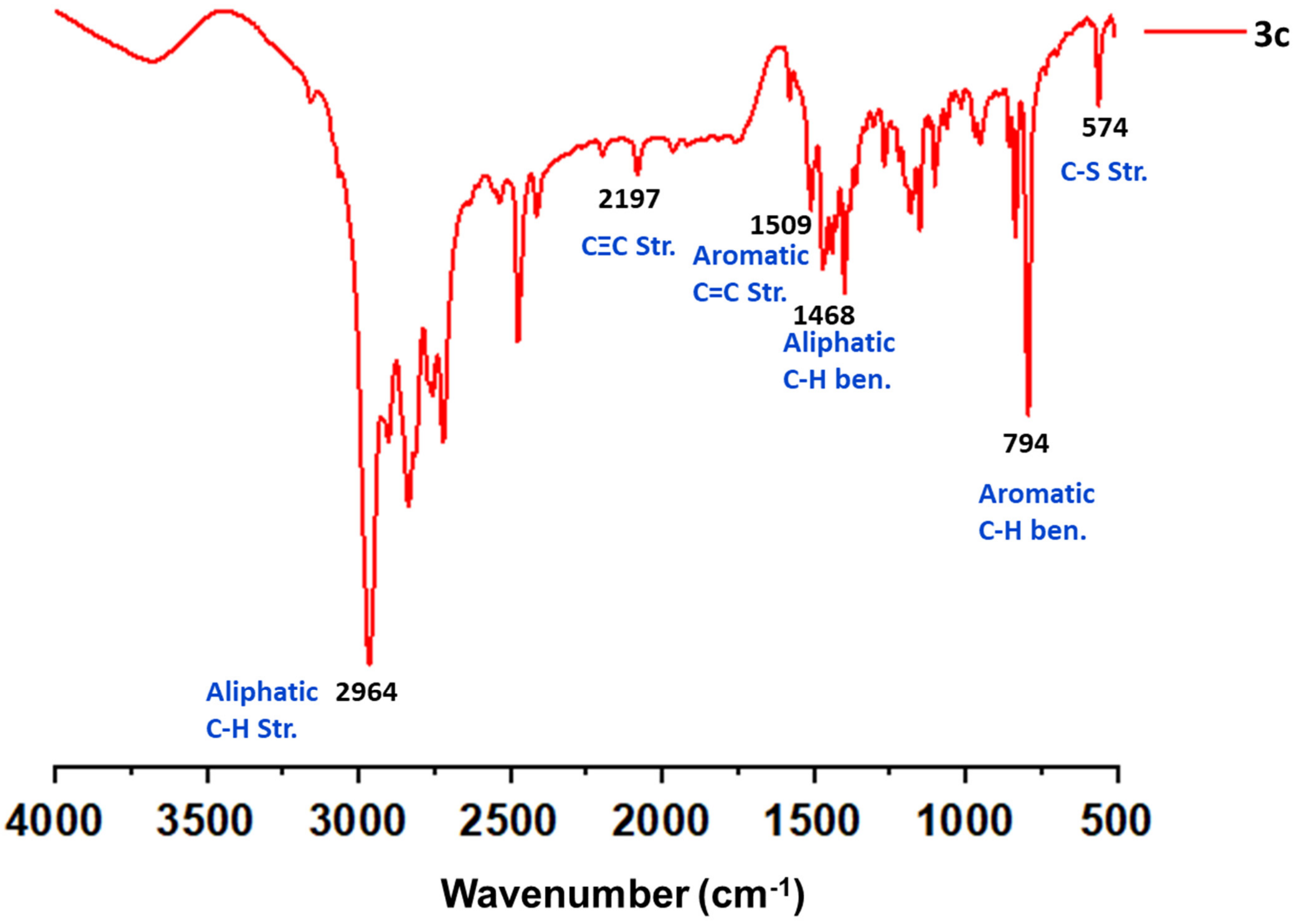

Figure S17 FTIR spectrum of **3c**

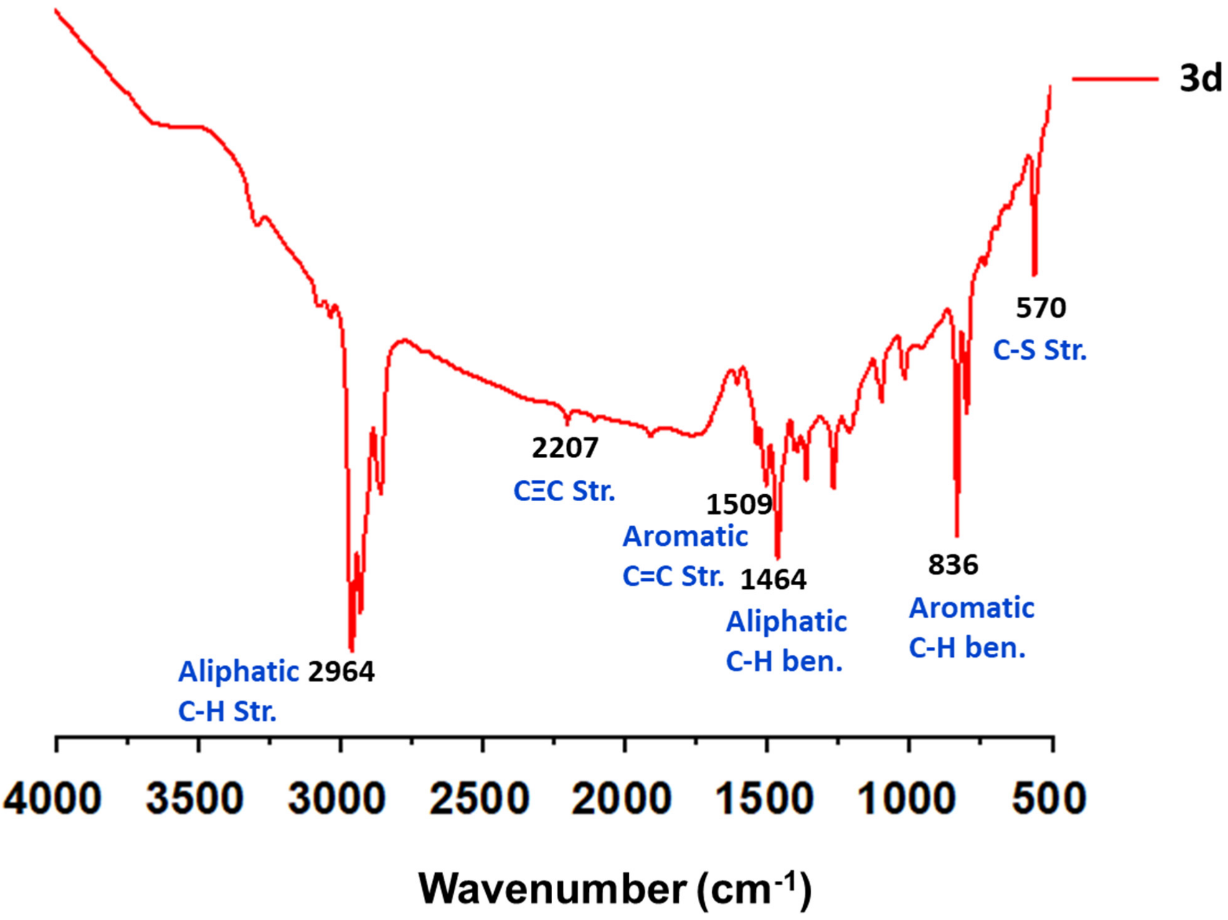

Figure S18 FTIR spectrum of **3d**

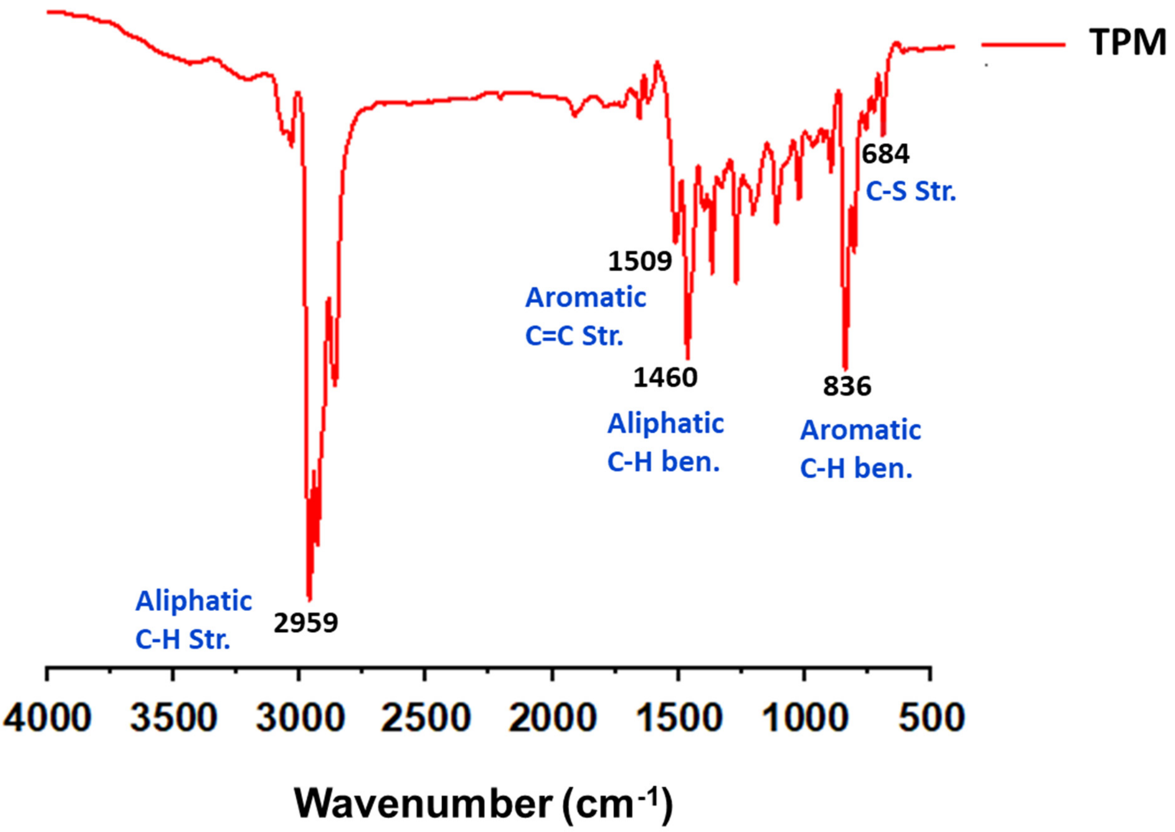

Figure S19 FTIR spectrum of **TPM**

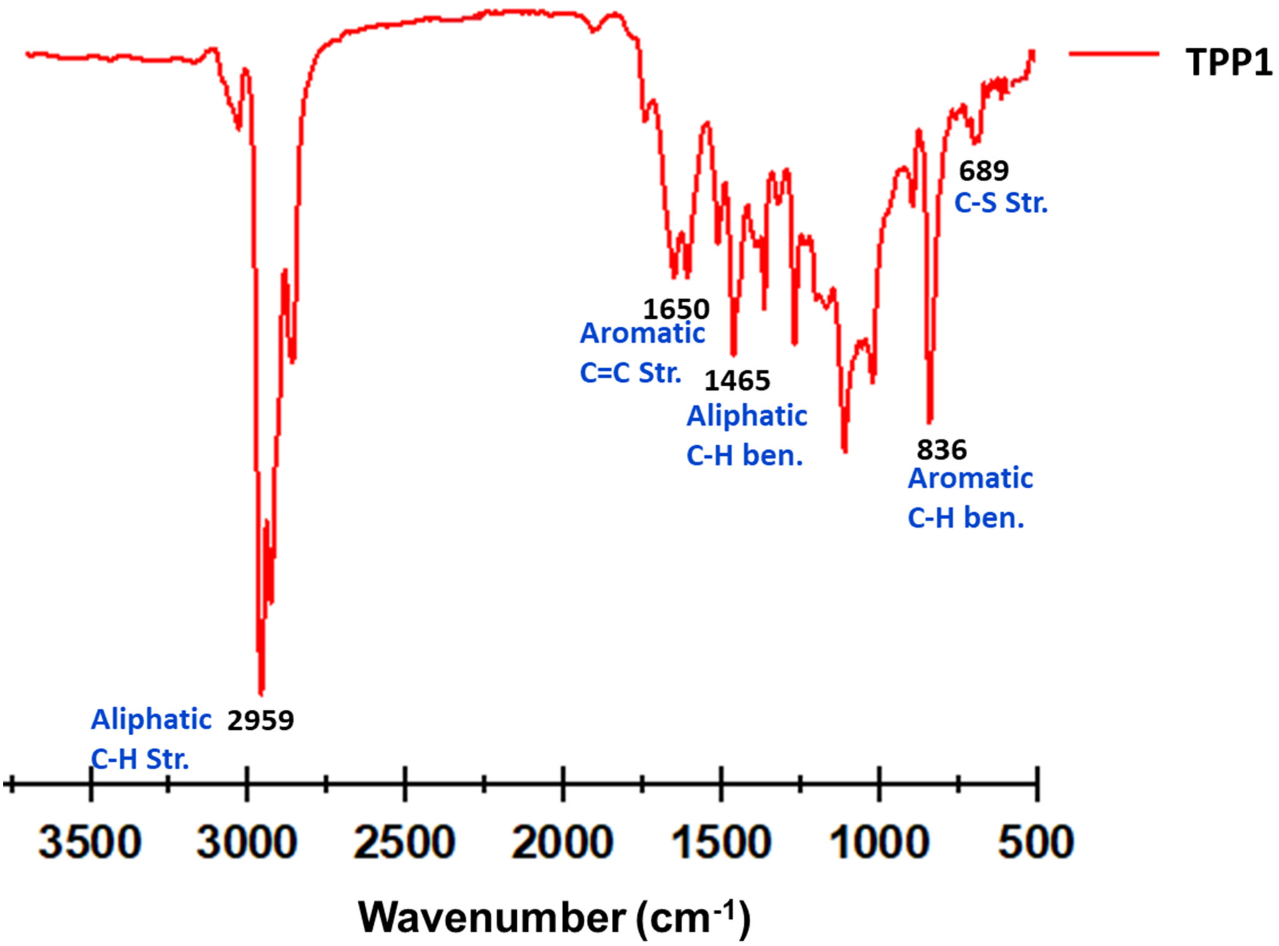

Figure S20 FTIR spectrum of **TPP1**

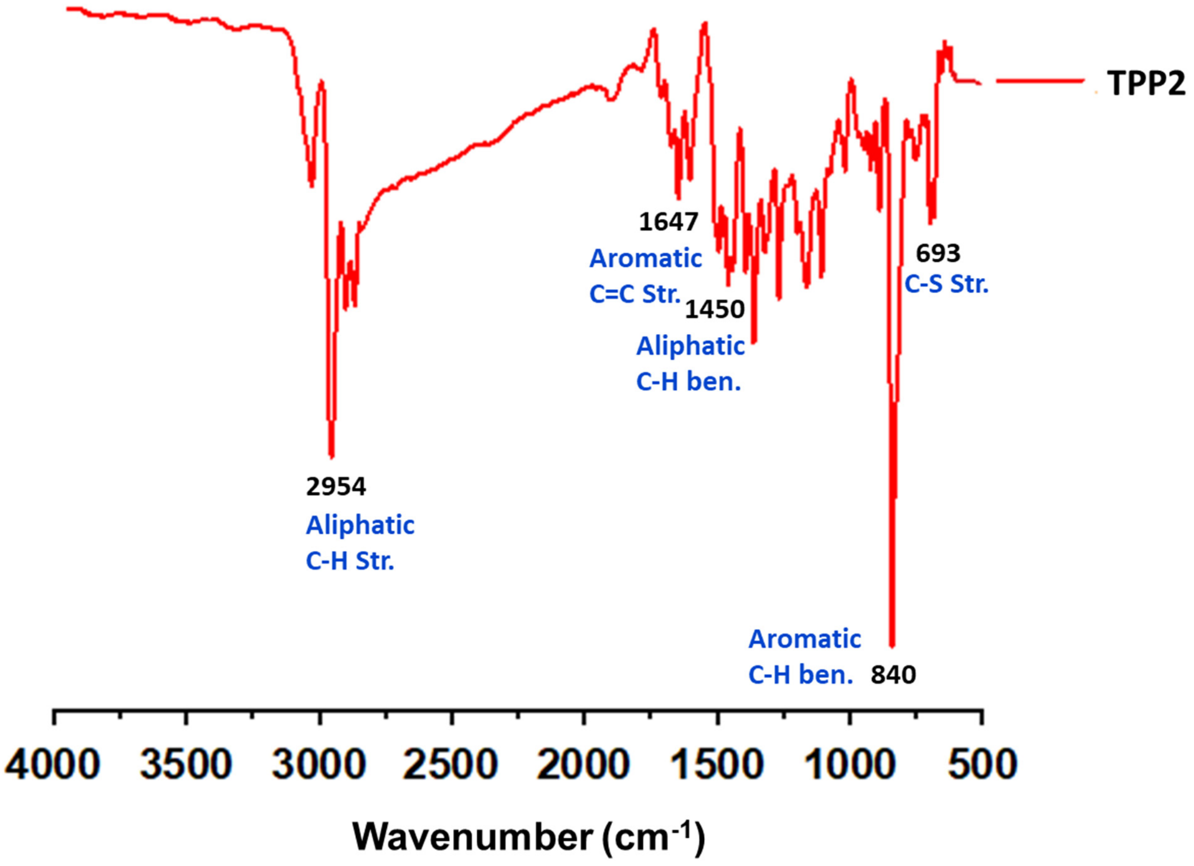

Figure S21 FTIR spectrum of **TPP2**

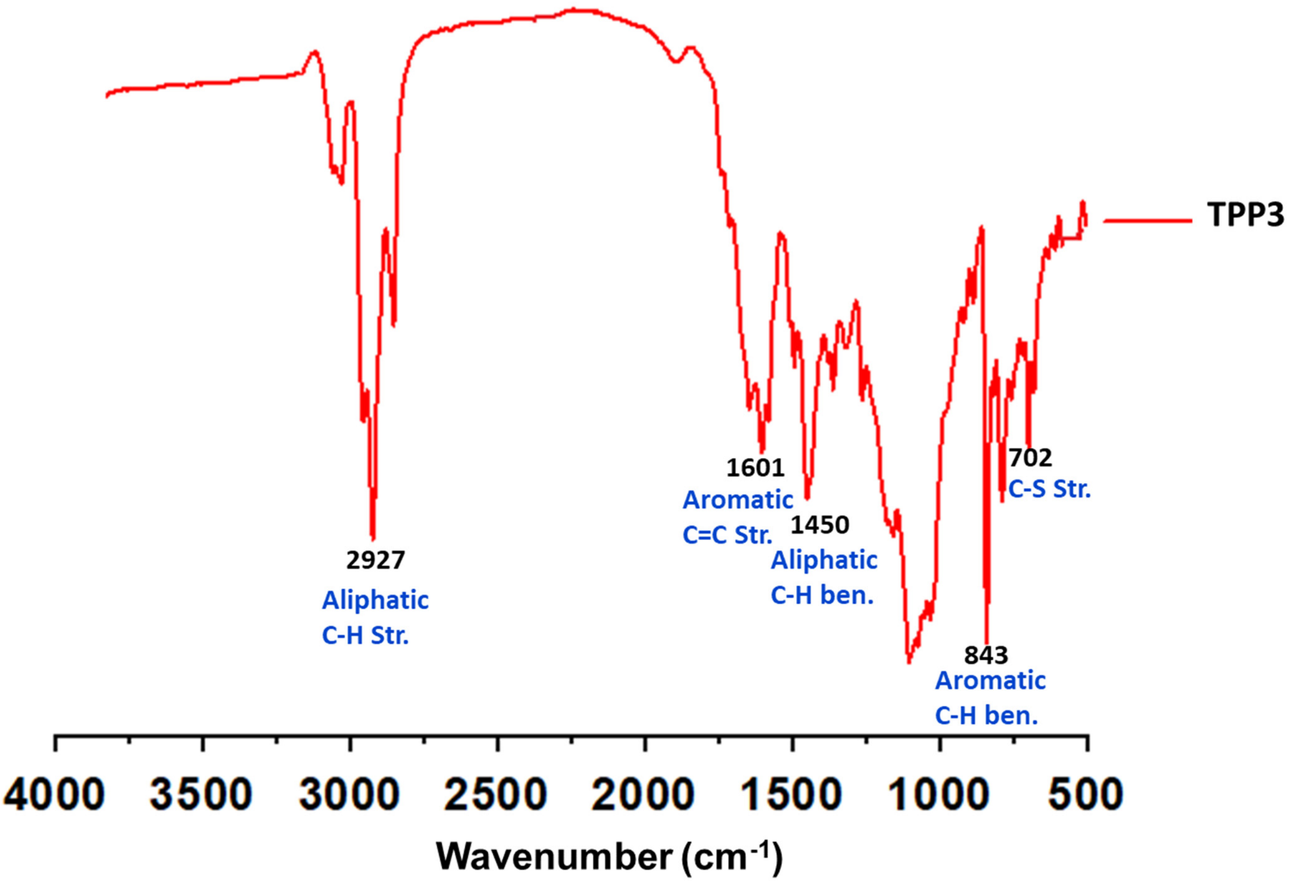

Figure S22 FTIR spectrum of **TPP3**

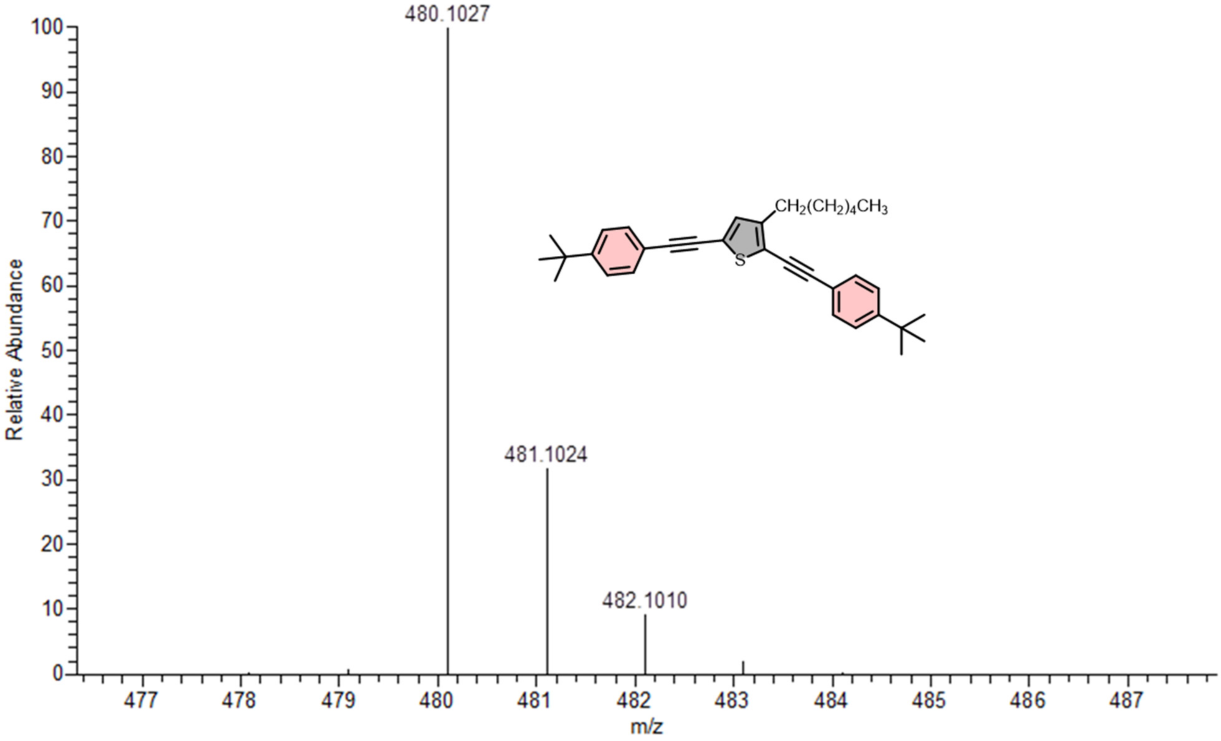

Figure S23 EI-HRMS spectrum of **3a**

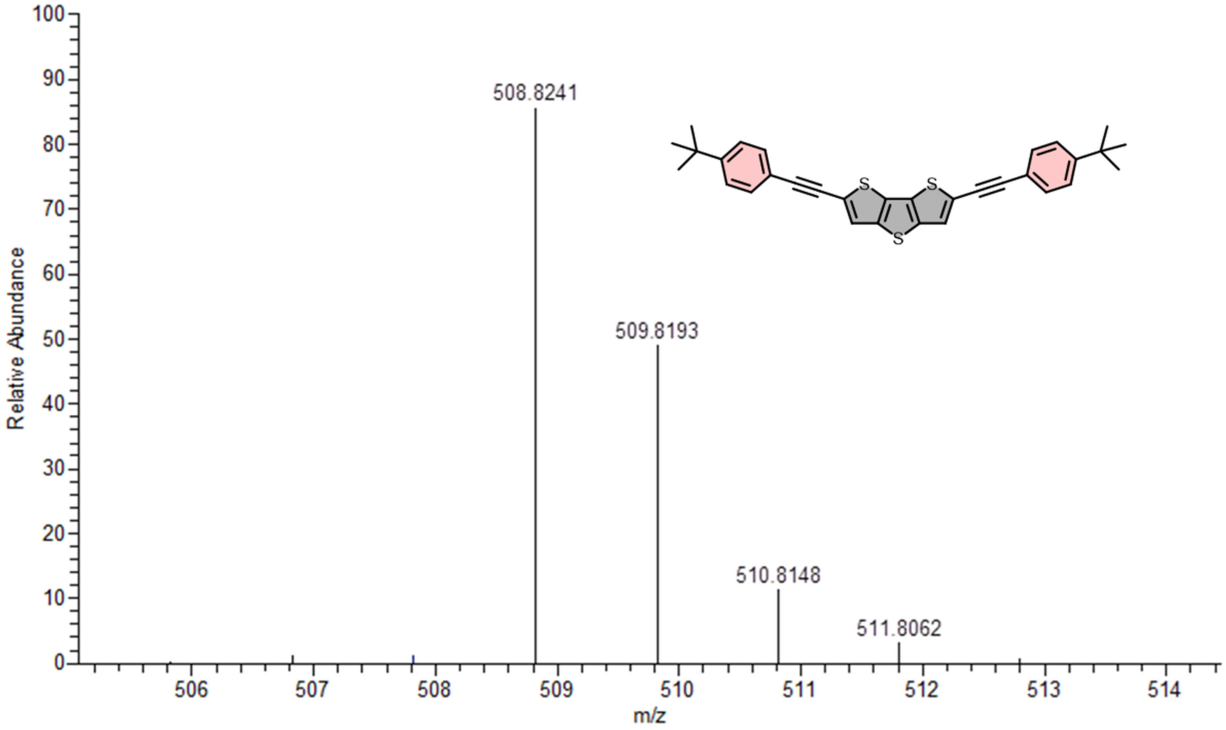

Figure S24 ESI-HRMS spectrum of **3b**

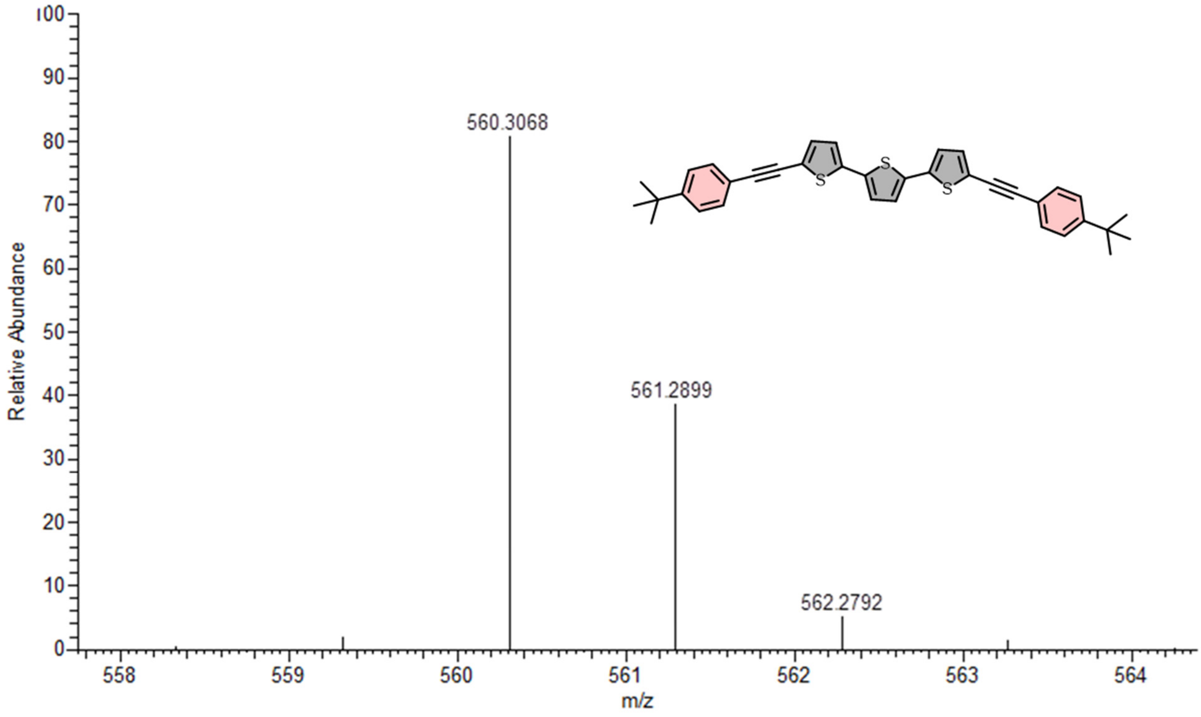

Figure S25 ESI-HRMS spectrum of **3c**

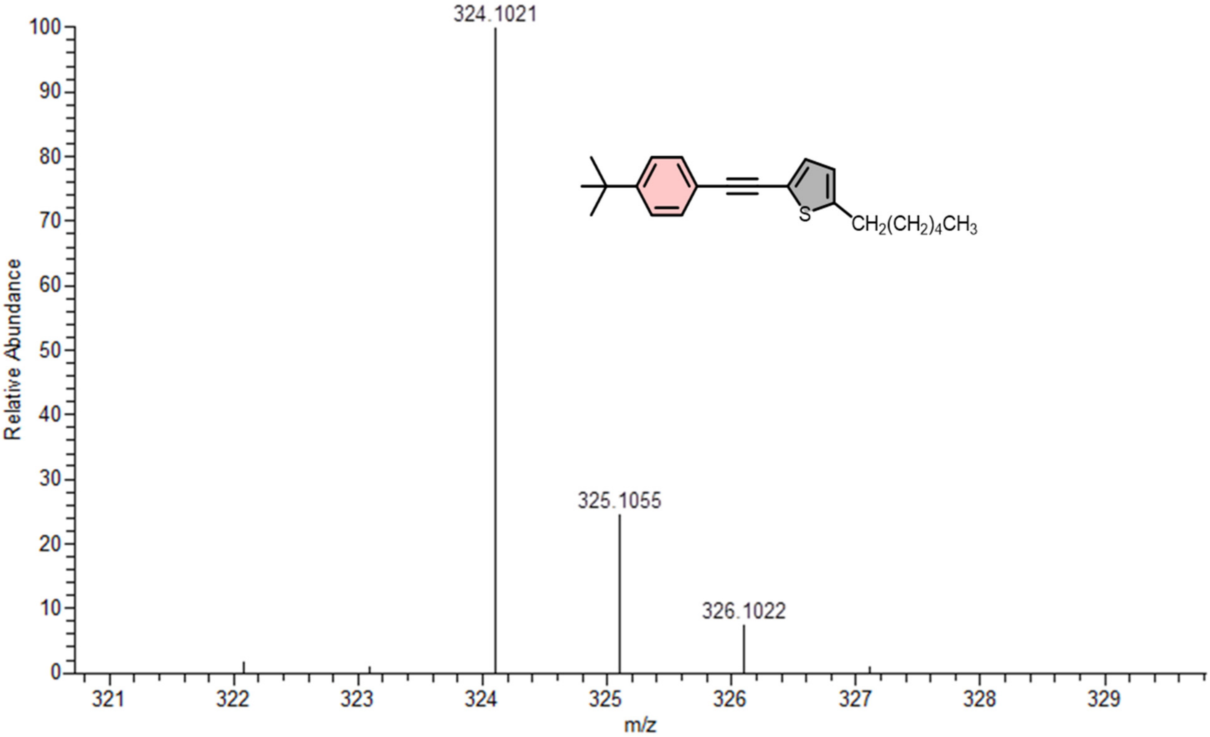

Figure S26 ESI-HRMS spectrum of **3d**

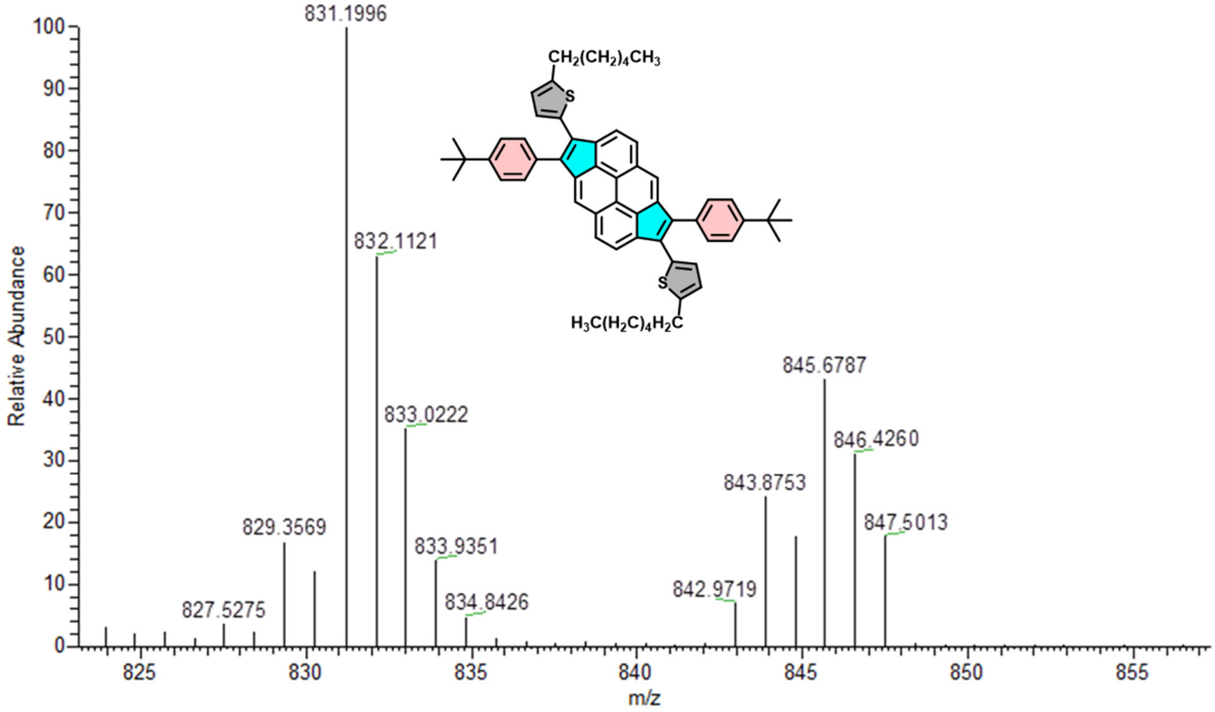

Figure S27 ESI-HRMS spectrum of **TPM**

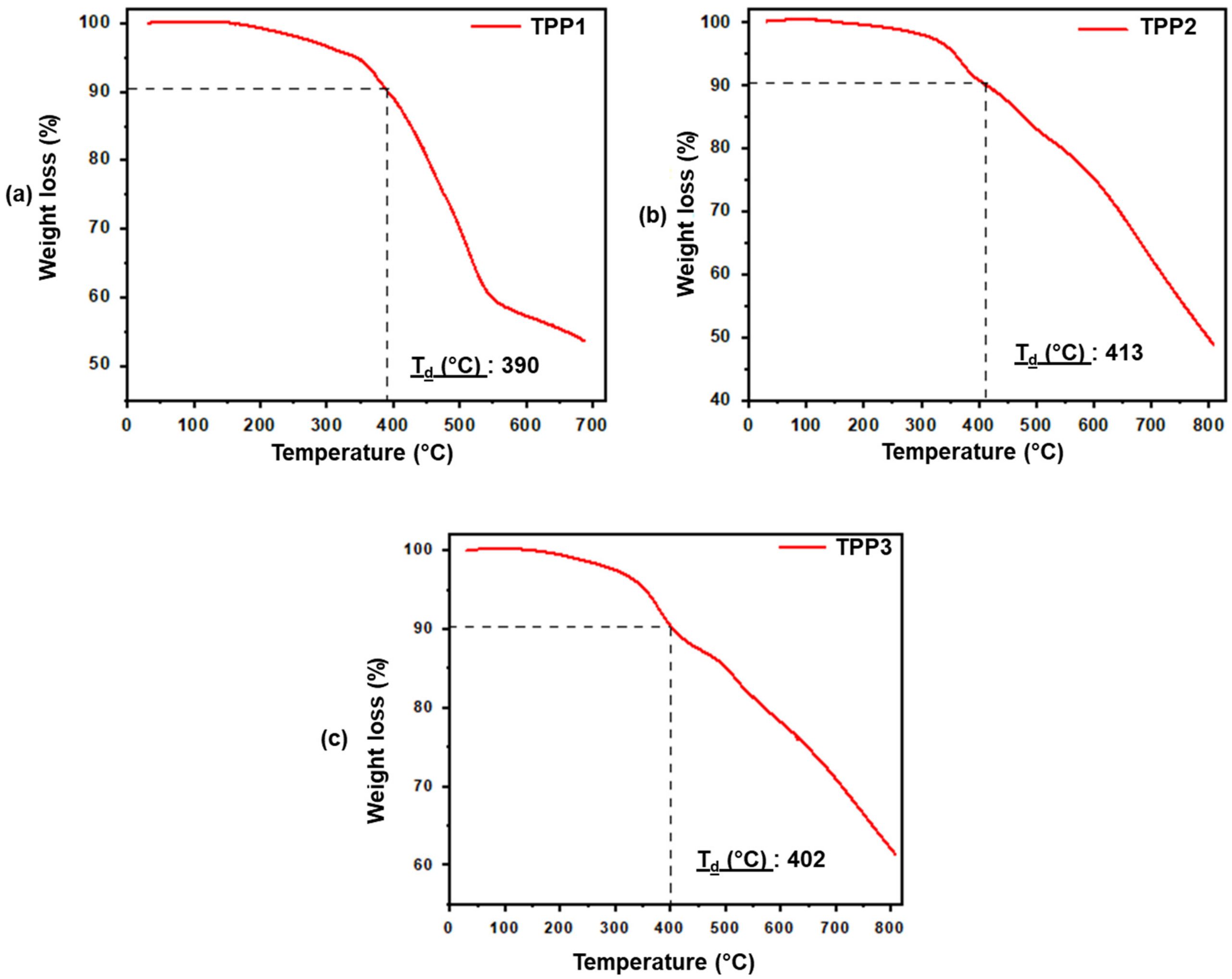

Figure S28 TGA thermograms of **(a) TPP1**, **(b) TPP2** and **(c) TPP3**, T_d represents the temperature of 10% weight loss

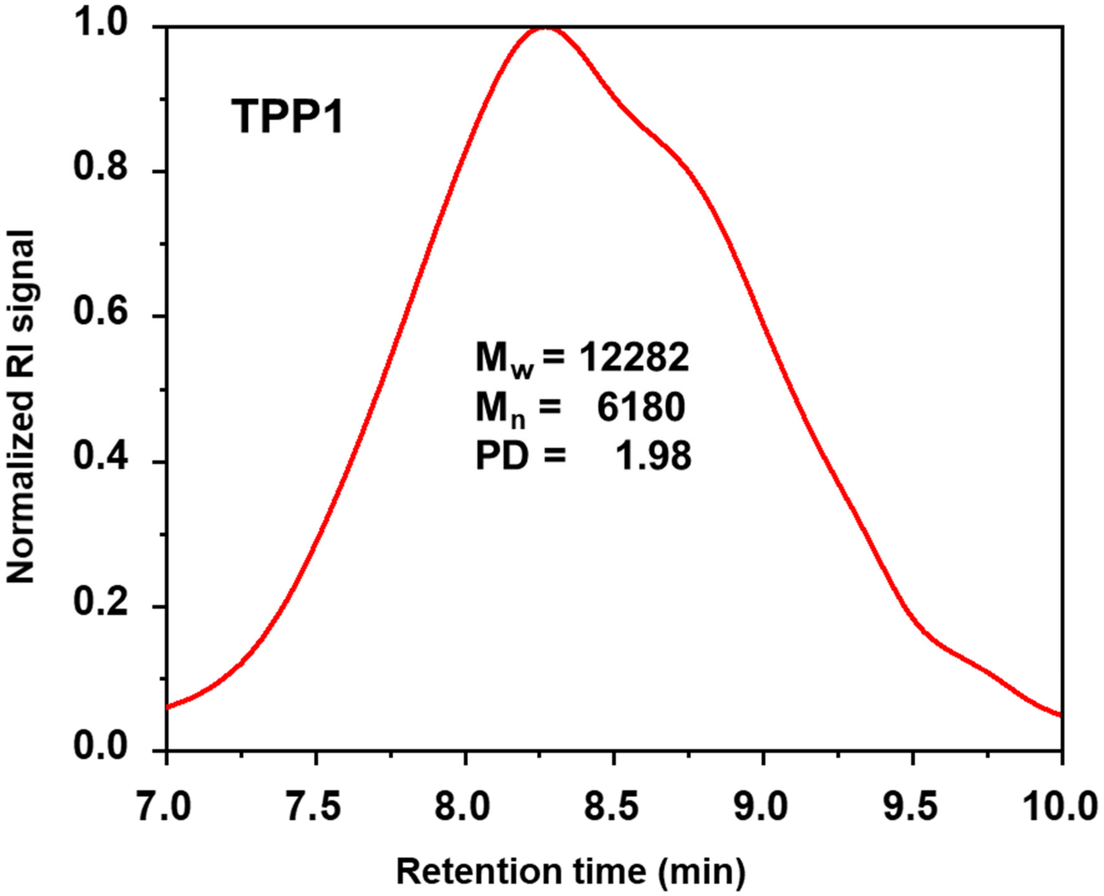

Figure S29 Normalized GPC chromatogram **TPP1**

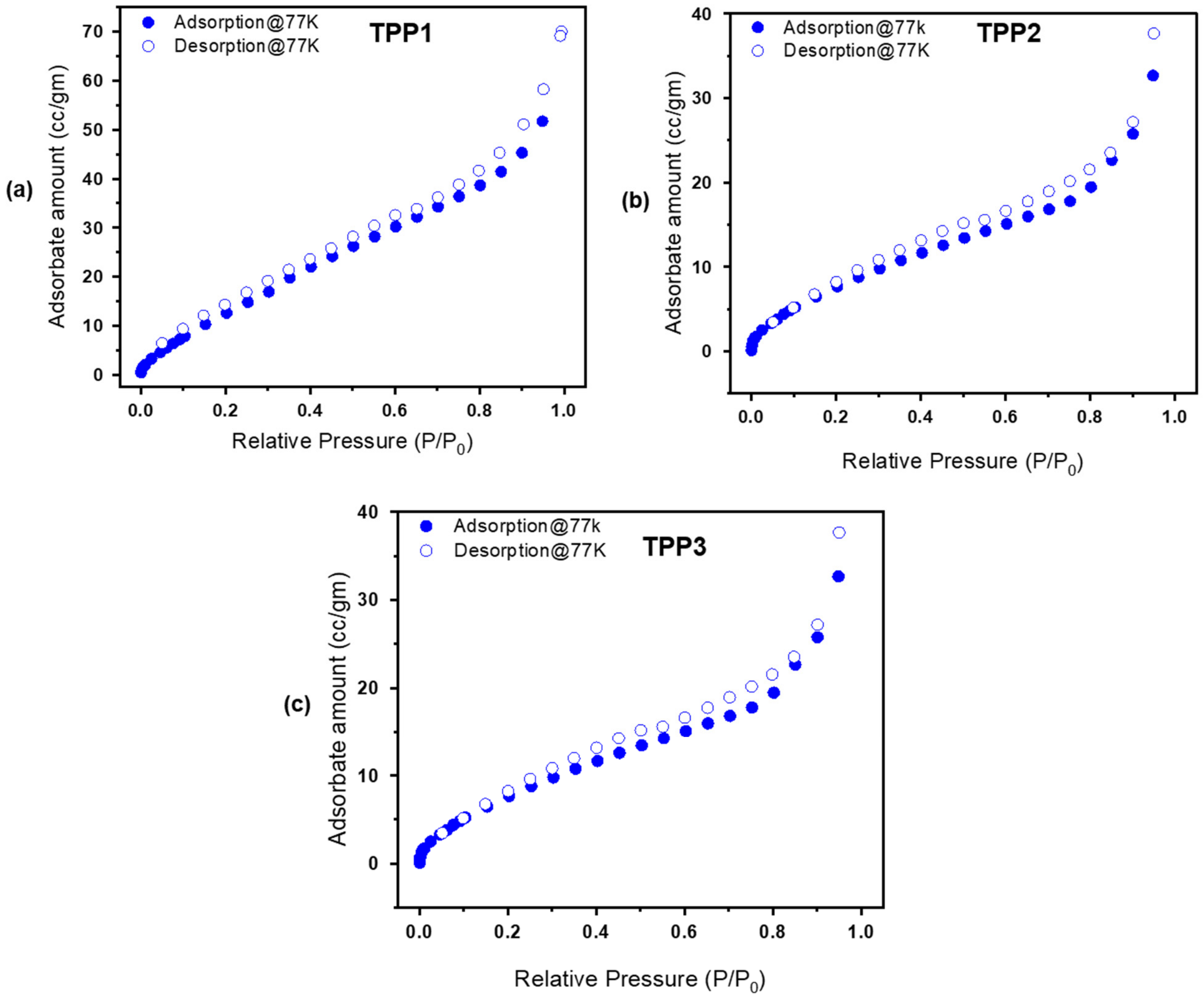

Figure S30 Nitrogen adsorption and desorption isotherms of (a) **TPP1**, (b) **TPP2** and (c) **TPP3** measured at 77 K**.**

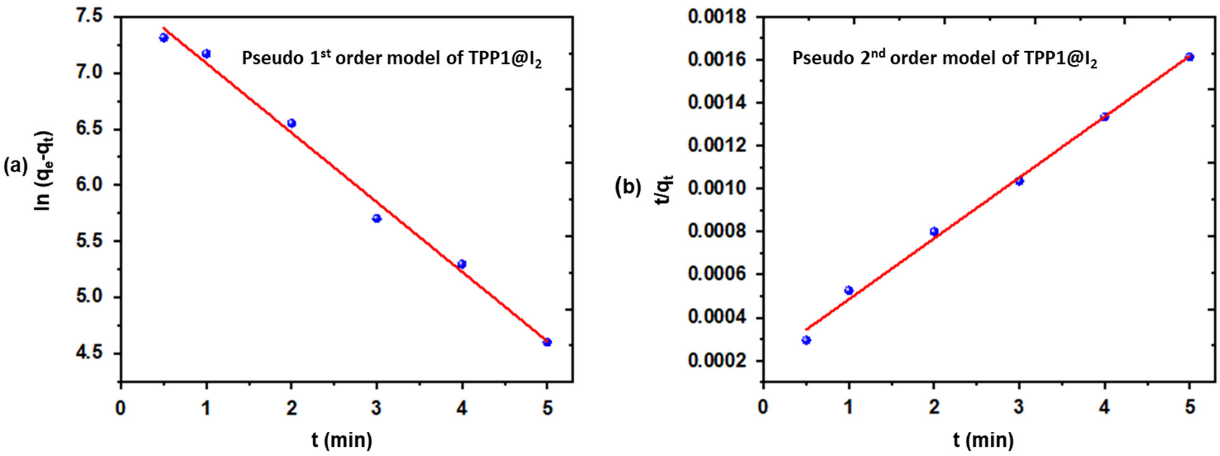

Polymer		Pseudo 1st order model			Pseudo 2nd order model		
	$q_{e,\,exp}$ (mg g^{-1})	$q_{e,\,cal}$ (mg g^{-1})	k_1 (min^{-1})	R^2	$q_{e,\,cal}$ (mg g^{-1})	k_2 (min^{-1})	R^2
**TPP1@I$_2$**	3200	2216	-0.02575	0.9901	3532	0.000396	0.99443

Figure S31 Pseudo-first-order **(a)** and pseudo-second-order **(b)** models of **TPP1@I$_2$**

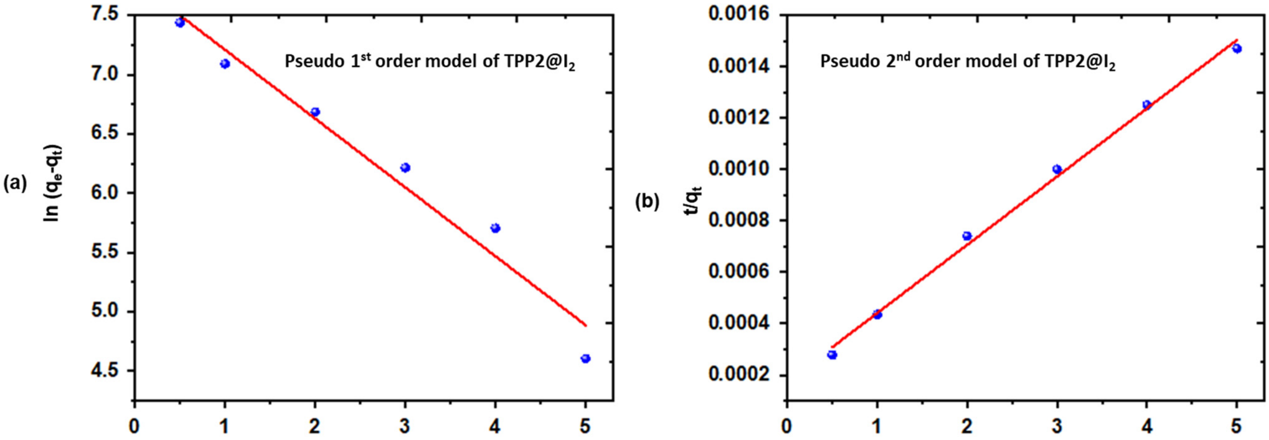

Polymer		Pseudo 1st order model			Pseudo 2nd order model		
	$q_{e,\,exp}$ (mg g^{-1})	$q_{e,\,cal}$ (mg g^{-1})	k_1 (min^{-1})	R^2	$q_{e,\,cal}$ (mg g^{-1})	k_2 (min^{-1})	R^2
**TPP2@I$_2$**	3500	2411	-0.02417	0.9566	3762	0.000402	0.9952

Figure S32 Pseudo-first-order **(a)** and pseudo-second-order **(b)** models of **TPP2@I$_2$**

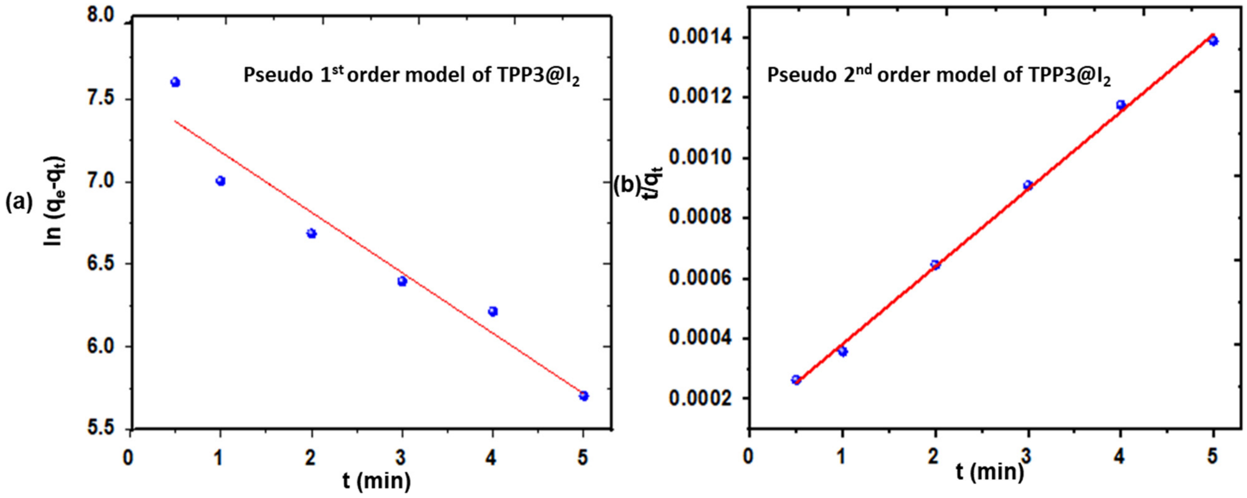

Polymer	Pseudo 1st order model				Pseudo 2nd order model		
	$q_{e, exp}$ (mg g^{-1})	$q_{e, cal}$ (mg g^{-1})	k_1 (min^{-1})	R^2	$q_{e, cal}$ (mg g^{-1})	k_2 (min^{-1})	R^2
**TPP3@I_2**	3900	1894	-0.01526	0.9278	3887	0.000528	0.9976

Figure S33 Pseudo-first-order **(a)** and pseudo-second-order **(b)** models of **TPP3@I_2**

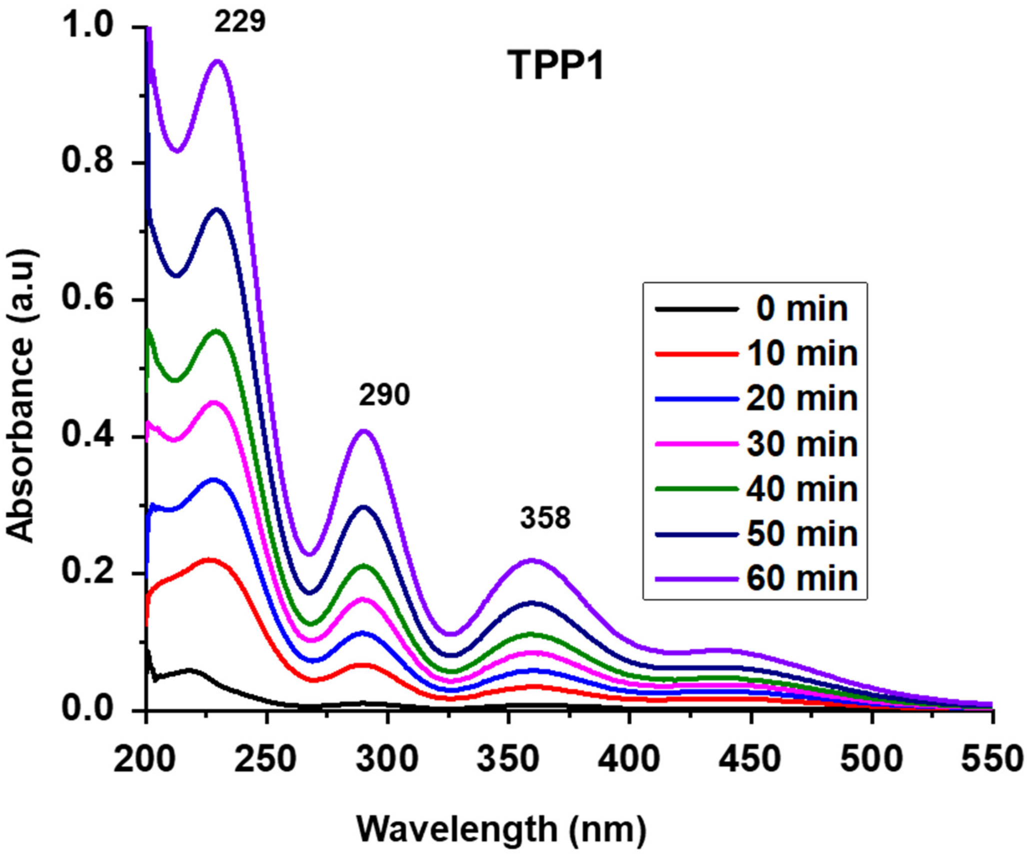

Figure S34 UV-Vis absorption spectra upon immersion of **TPP1@I_2** in ethanol

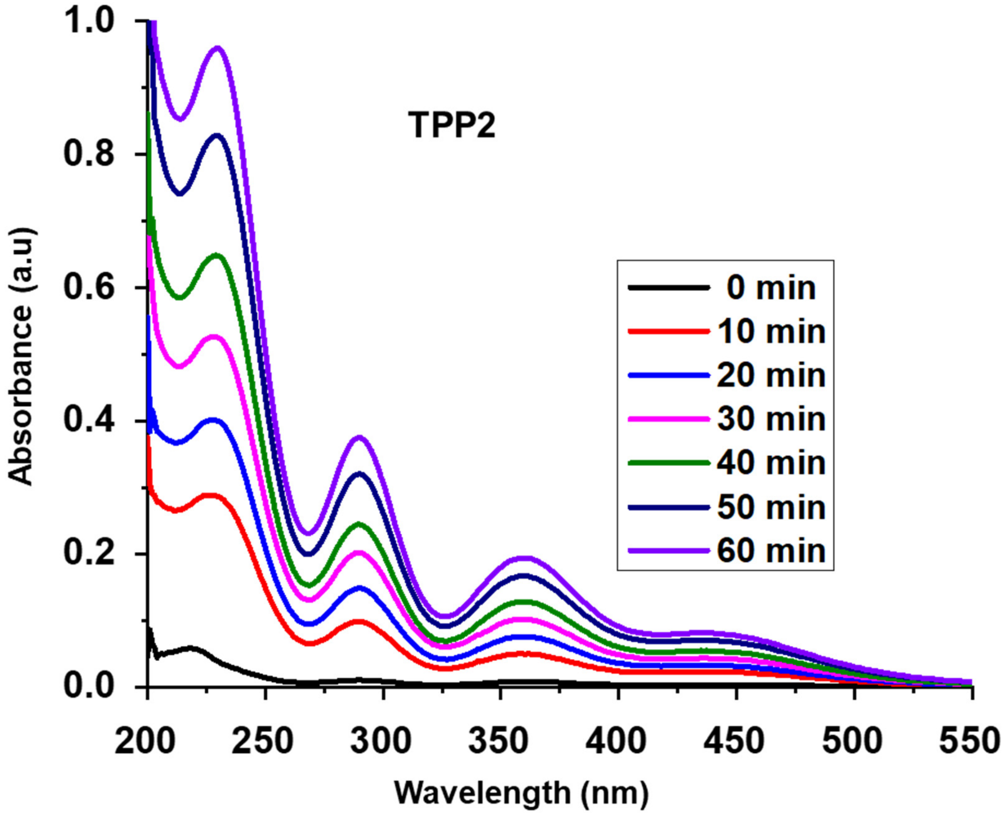

Figure S35 UV-Vis absorption spectra upon immersion of **TPP2@I$_2$** in ethanol

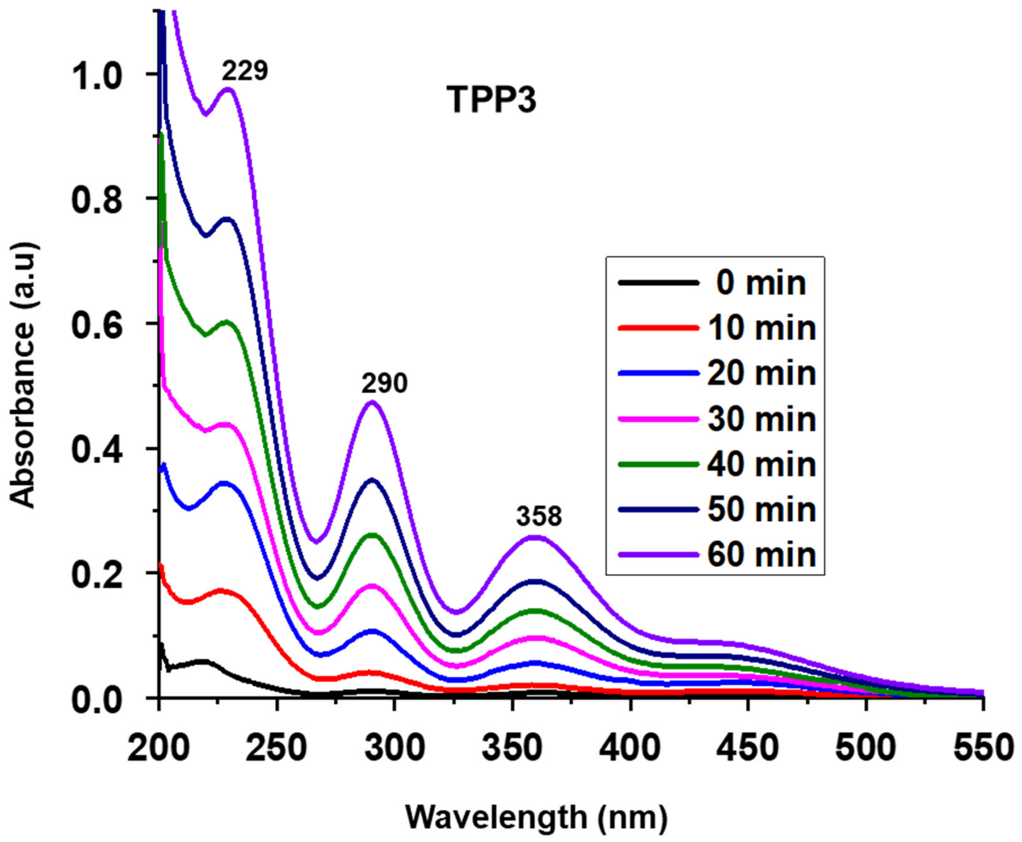

Figure S36 UV-Vis absorption spectra upon immersion of **TPP3@I$_2$** in ethanol
